# Supplementary material for: Outpacing conventional nicotinamide hydrogenation catalysis by a strongly communicating heterodinuclear photocatalyst
Source: Nat Commun. 2022 May 9;13:2538. doi: 10.1038/s41467-022-30147-4 (PMC9085789; doi:10.1038/s41467-022-30147-4)
Supplement: Supplementary file 1 — Supplementary Information [file 41467_2022_30147_MOESM1_ESM.pdf]

Supplementary Information

Outpacing conventional nicotinamide hydrogenation catalysis by a strongly communicating heterodinuclear photocatalyst

Linda Zedler[1,2,§], Pascal Wintergerst[3,§], Alexander K. Mengele[3], Carolin Müller[1,2], Chunyu Li[1,2], Benjamin Dietzek-Ivanšić [1,2,4,*], Sven Rau[3,*]

[1]Institute of Physical Chemistry, Friedrich Schiller University Jena, Helmholtzweg 4, 07743 Jena, Germany

[2]Leibniz Institute of Photonic Technology Jena, Department Functional Interfaces, Albert-Einstein-Straße 9, 07745 Jena, Germany

[3]Institute of Inorganic Chemistry I, Materials and Catalysis, Ulm University, Albert-Einstein-Allee 11, 89081 Ulm, Germany

[4]Center for Energy and Environmental Chemistry Jena (CEEC Jena), Philosophenweg 7a, 07743 Jena, Germany.

*e-mail: benjamin.dietzek@uni-jena.de ;sven.rau@uni-ulm.de

§ These authors contributed equally.

Table of Contents

Supplementary Methods S2
Supplementary Notes S10
Supplementary References S25

Supplementary Methods

NMR spectroscopy.

NMR spectra were recorded on a Bruker Avance III HD 400 or 500 at 293 K and processed with MestReNova software (Version 14.2.0). The chemical shifts δ are reported in parts per million (ppm). 1H NMR and 13C NMR shifts are referenced according to the applied deuterated solvent as internal standard. Constants J are presented as absolute values in Hz. For the characterization of the NMR signals the following abbreviations are used: s = singlet, d = doublet, t = triplet, q = quartet, m = multiplet and dd = doublet of doublets.

High resolution mass spectrometry.

High resolution mass spectrometry (HRMS) was performed on a Fourier Transform Ion Cyclotron Resonance (FT- ICR) mass spectrometer solariX (Bruker Daltonik) equipped with a 7.0 T superconducting magnet and interfaced to an Apollo II Dual ESI/MALDI source using trans-2-[3-(4-tert-butylphenyl)-2-methyl-2-propenylidene] malononitrile (DCTB) as a matrix.

Electrochemistry

Electrochemical data were obtained by cyclic voltammetry using a conventional single-compartment three-electrode cell arrangement in combination with a CH Instruments CHI 620E electrochemical workstation. A Pt wire was used as counter electrode, an Ag wire as quasi reference electrode and a glassy carbon electrode as the working electrode. The measurements were carried out in anhydrous and argon-saturated acetonitrile (ACN). Tetrabutylammonium hexafluorophosphate (0.1 M) was used as the supporting electrolyte at ambient temperature (20 ± 5 °C). All potentials are referenced to ferrocene/ferricenium [E(Fc/Fc^+) = 0.00 V].

Absorption and Emission spectroscopy.

Absorption spectra were recorded on a JASCO Spectrometer V-670. Continuous absorption spectra were recorded on a single-channel fiber-optic spectrometer (AvaSpec-ULS2048CL-EVO or AvaSpec-ULS2048XL). For illumination, a deuterium-halogen light source was used (AvaLight DH-S-BAL, Avantes Inc., USA). Emission spectroscopic investigations were performed with a JASCO Spectrofluorometer FP-8500. All samples were measured in quartz cuvettes with a path length of 10 mm.

Resonance Raman spectroscopy.

For rR spectroscopy, a single longitudinal mode diode laser at 405 nm (TopMode-405-HP, Toptica, Germany) was used for excitation. A grating spectrometer (IsoPlane 160, Princeton Instruments, USA) with an entrance slit width of 50 µm and 160 mm focal length was used for spectral detection employing diffraction gratings with 2400, 1200 and 600 grooves/mm as indicated in the figure captions. The laser power was attenuated to approximately 5 mW to reduce photodegradation of the analyte. The Raman scattering signals were collected in transmission and filtered from laser light with dielectric long-pass filters (Semrock, USA) before being focused on the entrance slit of the spectrograph. The Raman scattered light was spectrally dispersed, and the photons were detected by a thermoelectrically cooled CCD camera with 1340 x 100 pixels (PIXIS eXcelon, Princeton Instruments, USA). The band of the solvent ACN at 1375 cm^{-1} was used for normalizing the Raman intensities and calibrating the wavenumber scale. Spectral post-processing includes background correction, normalization, and subtraction of the solvent spectrum.

Transient absorption (TA) spectroscopy.

The femtosecond-TA data were recorded using a specially designed optical setup. For details see.[1,2] A regenerative Ti:sapphire amplifier (Libra, Coherent, USA) at 1 kHz pulse repetition rate was used for excitation. The output of the laser is split in two parts. The first fraction is focused into a rotating CaF_2

plate to generate a broadband white light supercontinuum. This broadband pulse is divided into reference and probe pulse. The other part of the laser output is used to generate the pump pulses at 400 and 470 nm of about 100 fs pulse duration. SHG of the fundamental in a nonlinear crystal is used for generation of the 400 nm pump pulse. The 470 nm pump pulse is generated in an optical parametric amplifier (Topas, Light conversion, Lithuania) by parametric conversion. A mechanical chopper is used to reduce the repetition rate of the pump pulses to 0.5 kHz and the polarization of pump and probe pulse is adjusted to the magic angle of 54.7° with respect to the white light probe beam using a Berek compensator and a polarizer. The probe pulse is focused into the 1 mm cuvette by a concave mirror of 500 mm focal length. The spectra of probe and reference pulses are recorded by a Czerny-Turner spectrograph with 150 mm focal length (SP2150, Princeton Instruments) equipped with a diode array detector (Pascher Instruments AB, Sweden). In the time range of 300 fs around time zero strong contributions of coherent artifact signals[3] are observed, which prevent the analysis of pump-probe data by multiexponential fitting algorithms with shorter time delay.
The TA data analysis first includes a spectral preprocessing step for chirp correction. Then a sum of exponential functions is fitted to the data by a least squares regression analysis using python software (python tool KiMoPack).[4] The pulse overlap range of ±150 fs is removed from the data analysis due to the coherent artifacts mentioned above. The amplitudes of the exponential fitting correspond to the decay associated spectra (DAS). For the investigation of the primary photoinduced processes, the sample was dissolved in anhydrous ACN or DCM (OD (400 nm) = 0.3 in a cell with 1 mm path length).

Nanosecond transient emission spectroscopy.

Nanosecond spectroscopy of transient emissions was used to study the lifetime of the long-lived species. The setup was used as described in the literature.[5] The pump pulses centered at 355 nm were generated by a continuum surelite Nd:YAG laser system (pulse duration 5 ns, repetition rate 10 Hz). A Continuum OPO Plus, pumped by a Continuum Surelite Nd:YAG laser, generated the pump pulses at 470 nm. The power of the pump beam was maintained at 0.2 mJ per pulse. The probe light is provided by a 75 W xenon arc lamp. Spherical concave mirrors are used to focus the probe beam into the samples and then send the beam to the monochromator (Acton, Princeton Instruments). The spectrally selected probe light is detected by a Hamamatsu R928 photomultiplier. Time-resolved emission spectra were recorded using a 475 nm long pass filter in front of the detector to eliminate pump scatter. The signal is amplified and processed by a commercially available detection system (Pascher Instruments AB). Each sample was freshly prepared, and its optical density was maintained at about 0.2 at the excitation wavelength. All measurements were performed in fluorescence cuvettes with 1 cm pathlength.
The stability of the samples during all spectroscopic experiments was checked by measuring the absorption spectra before and after each spectroscopic run.

All spectroscopic measurements were performed on individual sample solutions and the same sample solutions were not measured repeatedly.

Synthesis

All reactions were performed under air if not otherwise stated. All solvents utilized for synthetic purposes were obtained from VWR. They were of technical grade and were redistilled prior to use. Size exclusion chromatography was performed on a Sephadex (LH-20) column using methanol as mobile phase. All other chemicals were obtained from commercial sources. [(tbbpy)$_2$Ru(5-bromo-1,10-phenanthroline)](PF$_6$)$_2$[6], [(tbbpy)$_2$Ru(5-ethinyl-1,10-phenanthroline)](PF$_6$)$_2$[7], 5-ethinyl-1,10-

phenanthroline[7], 5-azido-1,10-phenanthroline[8] and [(tbbpy)$_2$Ru(tpphz)Rh(Cp*)Cl]Cl(PF$_6$)$_2$[9] were prepared according to literature procedures.

[(tbbpy)$_2$Ru(5-(5-[1,10-phenanthrolinyl]-ethynyl)-1,10-phenanthroline)]Cl$_2$ (1)

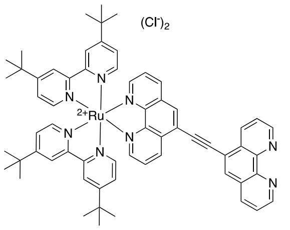

[(tbbpy)$_2$Ru(5-bromo-1,10-phenanthroline)](PF$_6$)$_2$ (150 mg, 126 µmol) and 5-ethinyl-1,10-phenanthroline (31 mg, 152 µmol) were dissolved in 10 mL THF and 2.5 mL diisopropylamine and degassed with argon for 20 minutes. Tetrakis(triphenylphosphine)palladium(0) (14.6 mg, 12.6 µmol) and CuI (1.2 mg, 6.3 µmol) were added to the solution and heated for 24 hours under reflux. The solvent was removed under vacuum and the crude product purified by filtration over aluminum oxide, by first washing with CH$_2$Cl$_2$ and then eluting with CH$_2$Cl$_2$/methanol (9/1, v/v). Afterwards, the solvent was removed under vacuum. The chloride salt was obtained by passing the compound through an ion exchange column (30 g Amberlite IRA-410, methanol). After size exclusion chromatography (Sephadex, methanol) and diffusion crystallization (ACN/MeOH/Et$_2$O), the pure compound was obtained as a red solid (55%, 75.6 mg, 69.3 µmol).

1H NMR (400 MHz, CD$_3$OD) δ 9.15 (dd, *J* = 4.1, 1.5 Hz, 1H), 9.14 (d, *J* = 1.5 Hz, 1H), 9.08 (dd, *J* = 4.4, 1.7 Hz, 1H), 9.04 (dd, *J* = 8.2, 1.7 Hz, 1H), 8.78 (d, *J* = 2.2 Hz, 2H), 8.75 – 8.70 (m, 3H), 8.57 – 8.49 (m, 2H), 8.29 (dd, *J* = 5.3, 1.3 Hz, 1H), 8.22 (dd, *J* = 5.3, 1.2 Hz, 1H), 8.02 (dd, *J* = 8.4, 5.2 Hz, 1H), 7.97 – 7.86 (m, 2H), 7.81 (dq, *J* = 7.4, 3.5 Hz, 3H), 7.65 – 7.55 (m, 4H), 7.40 (ddd, *J* = 6.2, 2.1, 1.1 Hz, 2H), 1.49 (m, 18H), 1.41 (s, 18H); 13C NMR (126 MHz, CD$_3$OD) δ 164.54, 164.40, 158.60, 158.41, 158.39, 154.01, 152.36, 152.29, 152.26, 152.22, 151.75, 149.18, 149.02, 146.99, 146.57, 138.15, 137.80, 136.34, 136.06, 133.90, 133.84, 131.98, 129.58, 129.30, 128.07, 127.93, 126.27, 126.15, 125.41, 122.85, 122.78, 122.67, 119.88, 94.80, 90.94, 36.70, 36.61, 30.64, 30.56. MALDI-HRMS (m/z): calcd. for [M – 2Cl]$^+$ ([$C_{62}H_{62}N_8Ru$]$^+$), 1020.41409; found, 1020.41380.

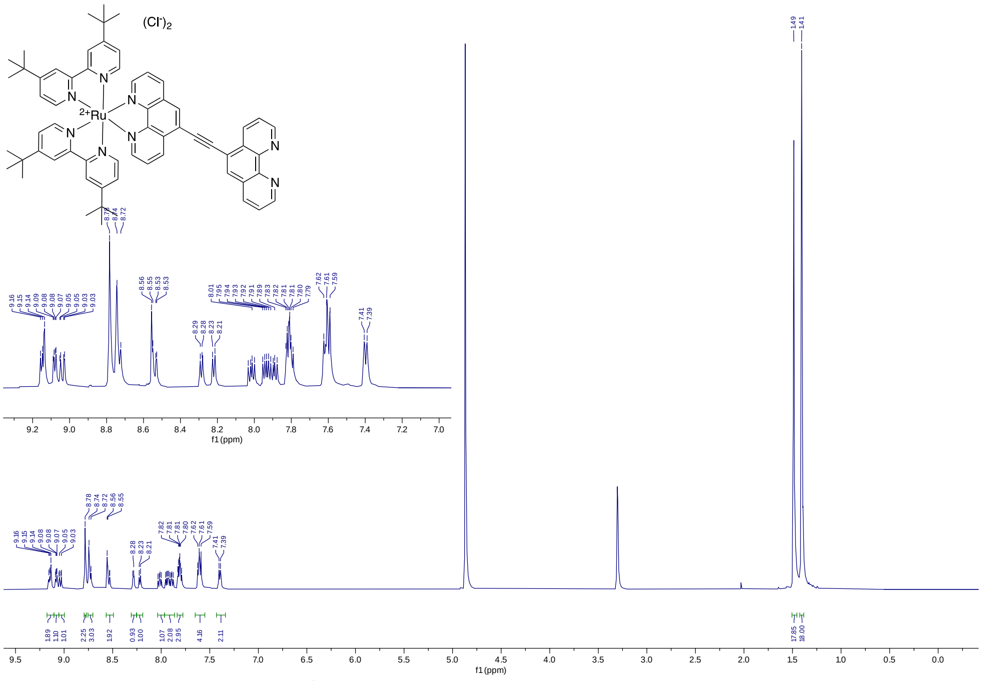

Supplementary Figure 1: ^{1}H-NMR spectrum of **1** in CD$_3$OD.

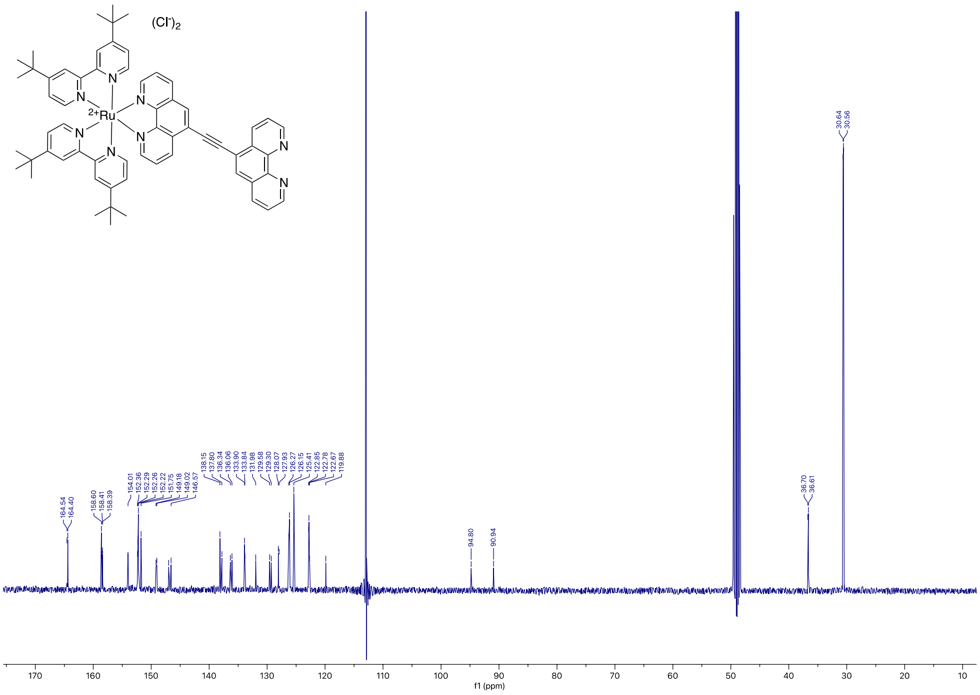

Supplementary Figure 2: ^{13}C-NMR spectrum of **1** in CD_3OD. Artifact at 110 ppm in the due to the UDEFT sequence used for acquisition.

[(tbbpy)$_2$Ru(5-(5-[1,10-phenanthrolinyl]-ethynyl)-1,10-phenanthroline)RhCp*Cl]Cl$_3$ (2)

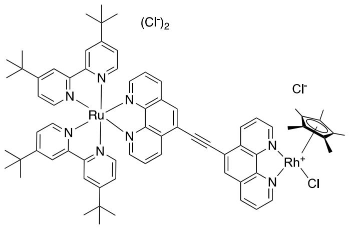

[(tbbpy)$_2$Ru((1,2-di(1,10-phenanthrolin-5-yl)ethyne)]Cl$_2$ (20.0 mg, 20.2 µmol) and [Rh(Cp*)Cl$_2$]$_2$ (6.9 mg, 12.1 µmol) were dissolved in 5 mL CH_2Cl_2. The mixture was stirred at rt for 16 hours. The solvent was removed under vacuum and the crude product purified via size exclusion chromatography (methanol, Sephadex). The dinuclear complex was obtained as a red powder (28 mg, 20 µmol, 96 %).

1H NMR (400 MHz, CD_3CN): δ 9.44 – 9.35 (m, 2H), 9.32 (d, *J* = 5.2 Hz, 1H), 9.19 (d, *J* = 8.3 Hz, 1H), 8.95 (d, *J* = 2.8 Hz, 1H), 8.89 (d, *J* = 8.3 Hz, 1H), 8.87 (d, *J* = 2.3 Hz, 1H), 8.73 (d, *J* = 8.1 Hz, 1H), 8.62 (d, *J* = 3.0 Hz, 2H), 8.58 (s, 2H), 8.35 (dd, *J* = 8.3, 5.2 Hz, 1H), 8.18 (dd, *J* = 10.5, 5.2 Hz, 2H), 8.12 (d, *J* = 5.3 Hz, 1H), 7.97 (dd, *J* = 8.3, 5.2 Hz, 1H), 7.82 (dd, *J* = 8.3, 5.4 Hz, 1H), 7.70 (dd, *J* = 6.3, 2.8 Hz, 2H), 7.49 – 7.43 (m, 4H), 7.26 – 7.20 (m, 2H), 1.76 (s, 15H), 1.46 (s, 9H), 1.45 (s, 9H), 1.37 (s, 18H); 13C NMR (126 MHz, CD_3OD): δ 163.15, 163.00, 157.18, 157.01, 156.99, 153.39, 153.21, 152.97, 152.92, 152.79, 152.74, 150.83, 147.83, 147.74, 145.45, 145.38, 145.27, 139.15, 138.99, 137.52, 136.57, 134.99, 133.33, 132.76, 130.50, 130.47, 130.10, 130.07, 127.53, 127.44, 127.31, 127.21, 126.84, 126.64, 124.91, 124.89, 124.76, 121.46, 121.39, 120.59, 120.18, 97.98, 97.91, 97.79, 97.73, 91.64, 91.34, 35.31, 35.22,

29.26, 29.18, 7.99, 7.70; MALDI-HRMS (m/z): [M-4Cl]$^+$ calcd. for $C_{72}H_{77}N_8RhRu$, 1258.43890; found 1258.44022; [M-4Cl-RhCp*]$^+$ calcd. for $C_{62}H_{62}N_8Ru$, 1020.41570; found, 1020.41425.

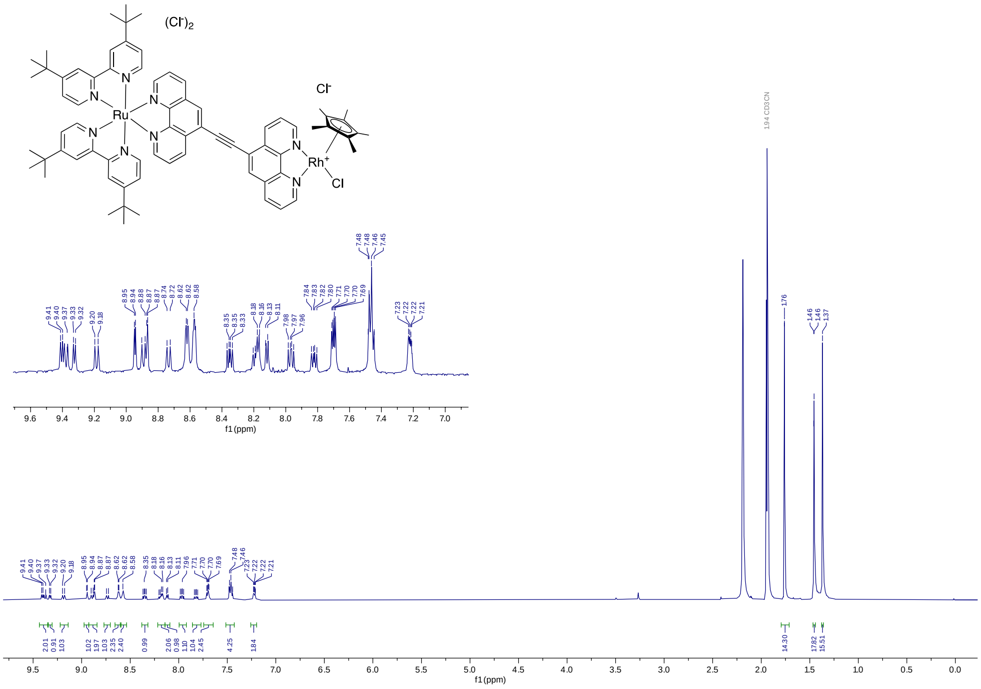

Supplementary Figure 3: ^{1}H-NMR spectrum of **2** in CD_3CN.

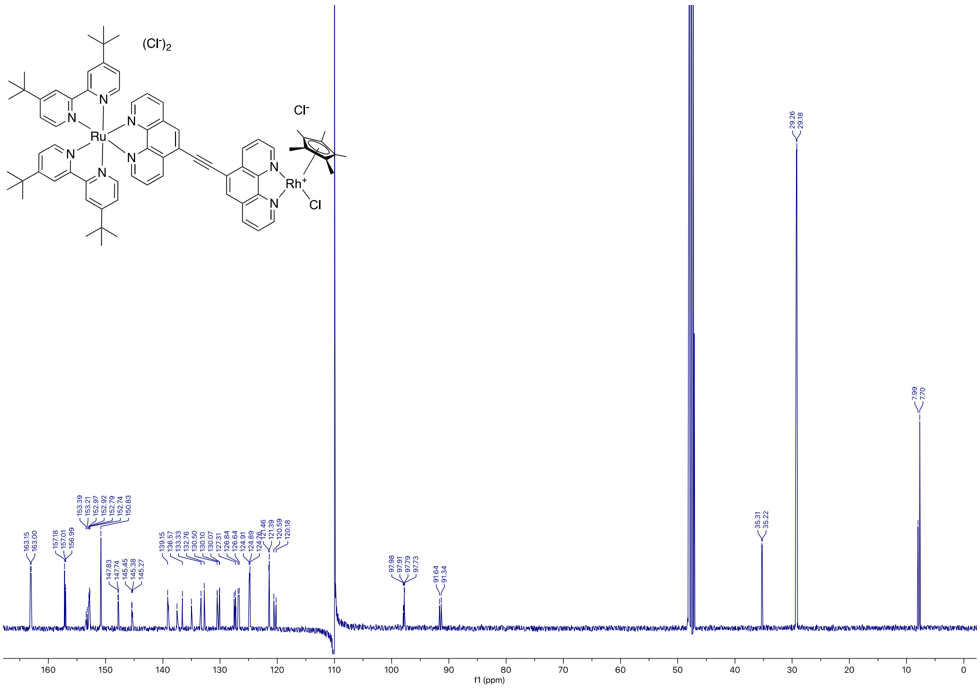

Supplementary Figure 4: ^{13}C-NMR spectrum of **2** in CD_3OD. Artifact at 110 ppm in the due to the UDEFT sequence used for acquisition.

[(tbbpy)$_2$Ru(5,5'-(1H-1,2,3-triazole-1,4-diyl)bis(1,10-phenanthroline))]Cl$_2$ (3)

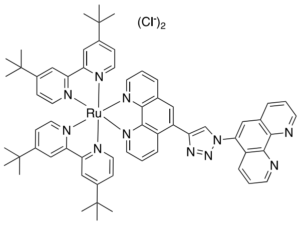

[(tbbpy)$_2$Ru(5-ethynyl-1,10-phenanthroline)](PF$_6$)$_2$ (150 mg, 133 µmol) and 5-azido-1,10-phenanthroline (43.9 mg, 199 µmol) were dissolved in 30 mL CH_2Cl_2. A solution of sodium ascorbate (210 mg, 1.06 mmol) and $CuSO_4$ (132 mg, 530 µmol) in 10 mL of water was added, and the mixture degassed by bubbling with argon for 10 minutes. After vigorous stirring for 16 h, potassium cyanide (173 mg, 2.66 mmol) was added and stirring continued for 1 h. The phases were separated, the organic phase was washed thoroughly with water and dried over Na_2SO_4. The solvent was removed under vacuum and the crude product purified by diffusion crystallization (ACN/Et_2O). The chloride salt was obtained by passing the compound through an ion exchange column (30 g Amberlite IRA-410, methanol). After removal of the solvent under vacuum, the complex was obtained as a red solid (105 mg, 82.9 µmol, 63 %).

1H NMR (400 MHz, CD_3OD): δ 9.57 (dd, J = 8.6, 1.2 Hz, 1H), 9.30 (d, J = 1.2 Hz, 1H), 9.26 (dd, J = 4.4, 1.6 Hz, 2H), 8.82 – 8.72 (m, 6H), 8.67 (dd, J = 8.2, 1.7 Hz, 1H), 8.47 (s, 1H), 8.43 (dd, J = 8.4, 1.6 Hz, 1H), 8.28 (dd, J = 5.2, 1.2 Hz, 1H), 8.23 (dd, J = 5.2, 1.2 Hz, 1H), 7.99 – 7.86 (m, 4H), 7.84 – 7.78 (m, 2H), 7.64 – 7.55 (m, 4H), 7.41 – 7.36 (m, 2H), 1.49 (s, 18H), 1.41 (s, 9H), 1.41 (s, 9H); 13C NMR: (126 MHz, CD_3OD): δ 164.50, 164.37, 158.62, 158.59, 158.45, 153.66, 153.54, 152.79, 152.21, 152.18, 152.14, 149.67, 148.97, 147.08, 146.86, 145.90, 138.86, 137.99, 137.03, 133.46, 133.06, 131.93, 130.83, 130.01, 129.57, 129.00, 128.77, 127.82, 127.52, 126.27, 126.24, 126.11, 126.01, 125.92, 125.70, 125.53, 122.86, 122.83, 122.78, 122.69, 36.70, 36.61, 30.64, 30.56; MALDI-HRMS (m/z): $[M\text{-}Cl]^+$ calcd. for $[C_{62}H_{63}ClN_{11}Ru]^+$, 1208.39587; found, 1208.39305.

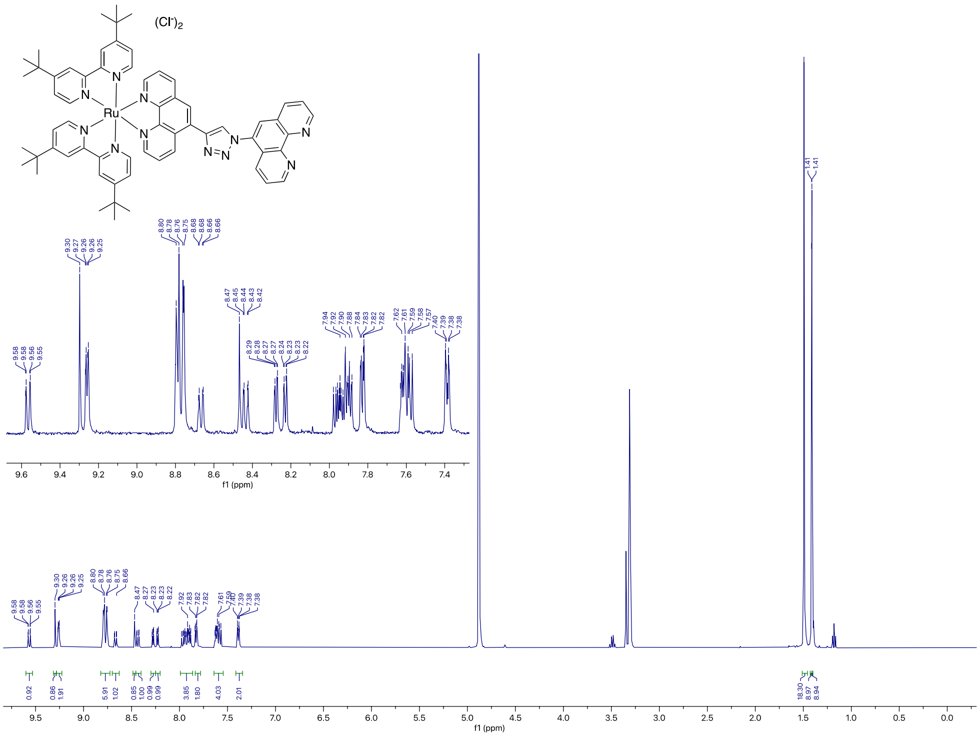

Supplementary Figure 5: ^{1}H-NMR spectrum of **3** in CD_3OD.

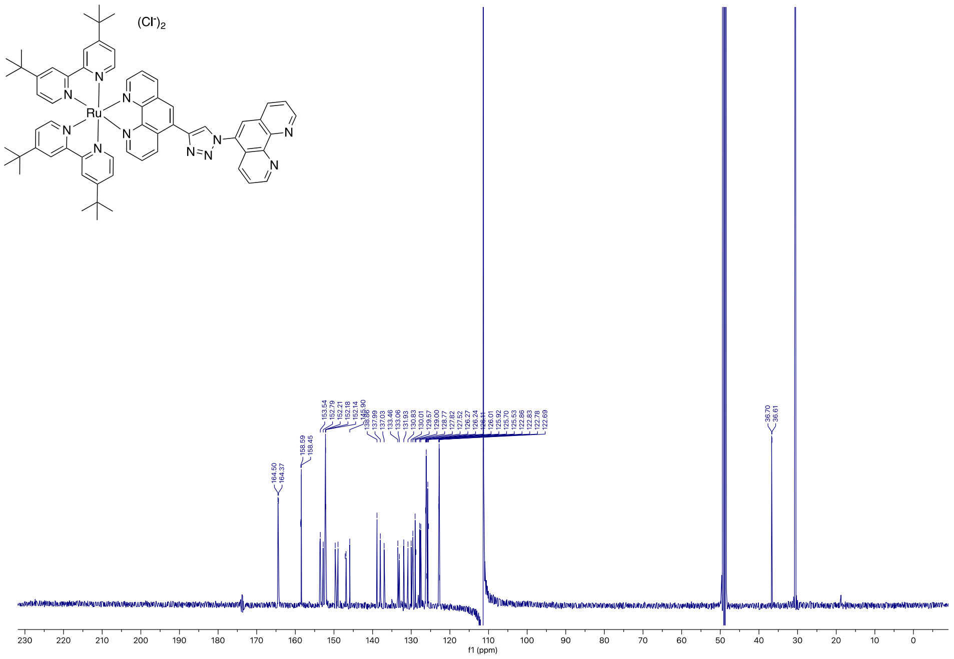

Supplementary Figure 6: ^{13}C-NMR spectrum of **3** in CD_3OD. Artifact at 110 ppm in the due to the UDEFT sequence used for acquisition.

[(tbbpy)$_2$Ru(5,5'-(1H-1,2,3-triazole-1,4-diyl)bis(1,10-phenanthroline))RhCp*Cl]Cl$_3$ (4)

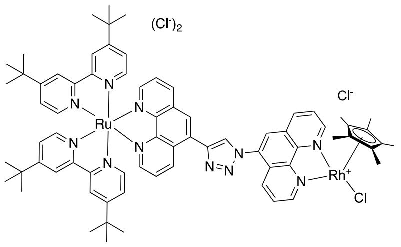

[(tbbpy)$_2$Ru(5,5'-(1H-1,2,3-triazole-1,4-diyl)bis(1,10-phenanthroline))]Cl$_2$ (25.0 mg, 22.0 µmol) and [Rh(Cp*)Cl$_2$]$_2$ (7.5 mg, 12.1 µmol) were dissolved in 5 mL DCM. The mixture was stirred at 40 °C for 3 hours. The solvent was removed under vacuum and the crude product purified via size exclusion chromatography (methanol, Sephadex). The dinuclear complex was obtained as a red powder (31 mg, 21 µmol, 96 %).

1H NMR (500 MHz, CD_3OD): δ 9.62 – 9.54 (m, 3H), 9.47 (d, J = 1.1 Hz, 1H), 9.10 (dd, J = 8.1, 3.2 Hz, 1H), 9.08 – 8.99 (m, 1H), 8.87 (s, 2H), 8.86 – 8.74 (m, 6H), 8.40 – 8.34 (m, 2H), 8.32 (dd, J = 5.2, 1.2 Hz, 1H), 8.28 (dd, J = 5.3, 1.3 Hz, 1H), 8.05 – 7.92 (m, 2H), 7.90 – 7.85 (m, 2H), 7.68 – 7.62 (m, 4H), 7.47 – 7.41 (m, 2H), 1.90 (s, 15H), 1.54 (s, 18H), 1.45 (s, 9H), 1.45 (s, 9H); 13C NMR (126 MHz, CD_3OD) δ 164.46, 164.33, 158.59, 158.56, 158.43, 154.85, 154.62, 153.72, 153.58, 152.23, 152.20, 152.15, 149.63, 149.00, 147.31, 146.80, 146.15, 141.33, 138.05, 137.12, 136.97, 134.11, 131.90, 130.77, 130.65, 129.81, 129.76, 129.03, 128.95, 128.88, 127.84, 127.81, 127.59, 126.27, 126.24, 126.11, 125.92, 122.84, 122.82, 122.77, 99.29, 99.23, 36.69, 36.60, 30.65, 30.57, 9.09; MALDI-HRMS (m/z): [M-Cl]$^+$ calcd. for [$C_{72}H_{78}Cl_3N_{11}RhRu$]$^+$, 1408.36090; found, 1408.35948; [M-4Cl]$^+$ calcd. for [$C_{72}H_{78}N_{11}RhRu$]$^+$, 1301.45590; found 1301.45369; [M-4Cl-RhCp*Cl]$^+$ calcd. for [$C_{62}H_{63}N_{11}Ru$]$^+$, 1063.43270, found, 1063.43058.

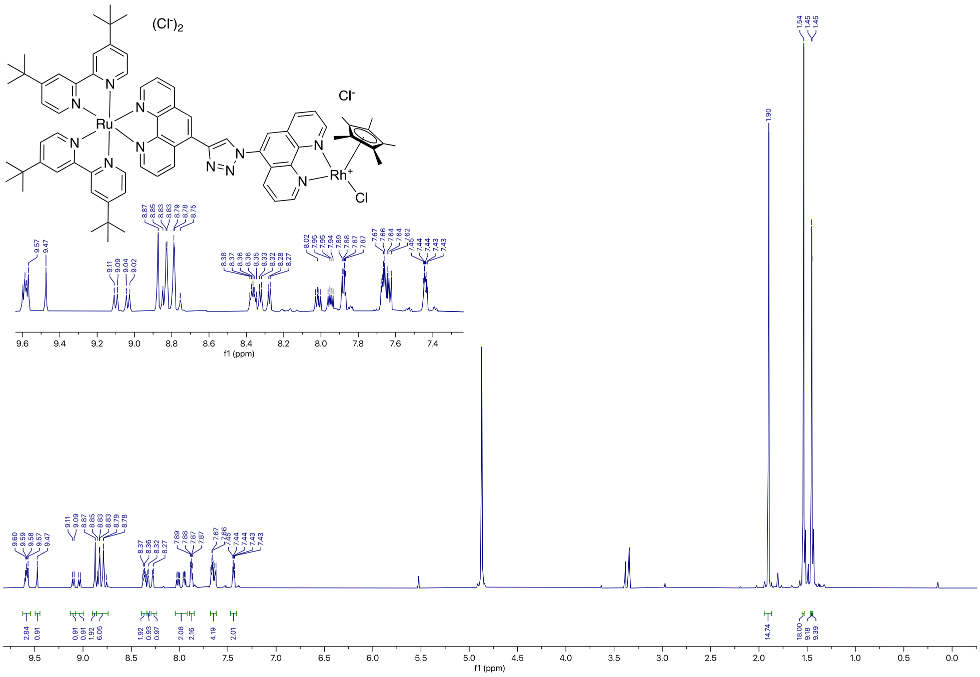

Supplementary Figure 7: ^{1}H-NMR spectrum of **4** in CD_3OD.

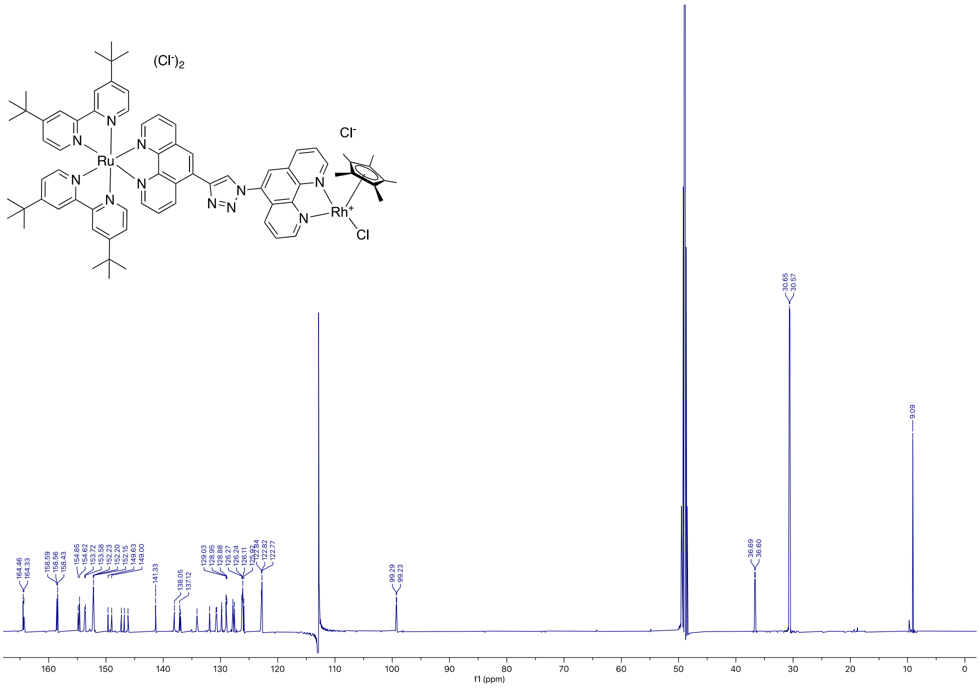

Supplementary Figure 8: ^{13}C-NMR spectrum of **4** in CD_3OD. Artifact at 110 ppm in the due to the UDEFT sequence used for acquisition.

Supplementary Notes

UV/vis absorption and emission data

Supplementary Table 1: Absorption and emission maxima of complexes **1**-**4**. The optical density at the MLCT absorption was set to 0.1. (λ_{exc} = 450 nm).

	solvent	$\lambda_{MLCTmax\ abs}$ / nm	ε_{MLCT} / L mol^{-1}cm^{-1}	$\lambda_{max\ emi}$ / nm	emission intensity / arb. u.	$\Phi_{deaerated}$	$\Phi_{aerated}$	$\tau_{deaerated}$/ns
**1**	ACN	454	19300	625	348	0.0921	0.0074	124
	DCM	458	16400	615	1320			
**2**	ACN	454	18600	627	107	0.0078	0.0013	136
	DCM	458	19300	611	243			
**3**	ACN	453	16400	617	523	0.1549	0.0113	114
	DCM	457	17000	606	1850			
**4**	ACN	452	18000	620	436	0.1149	0.0108	113
	DCM	457	18200	600	1620			

Electrochemistry

Supplementary Table 2: Reduction and oxidation waves obtained from cyclic voltammetry (ACN, 0.1 M (nBu)$_4$NPF$_6$, 0.5 mM complex).

E / V vs. Fc$^+$/Fc	E_{red1}(Rh)	E_{red2}(phen)	E_{red3}(bpy)	E_{red4}(bpy)	E_{ox1} (Ru)
**1**		-1.57	-1.92	-2.17	0.86
**2**	-1.16	-1.59	-1.95	-2.18	0.83
**3**		-1.70	-1.98	-2.23	0.80
**4**	-1.13	-1.69	-1.98	-2.25	0.80
**5**[10]	-1.09	-1.44	-1.86	-2.07	0.86

Thermal Catalysis

Mechanism

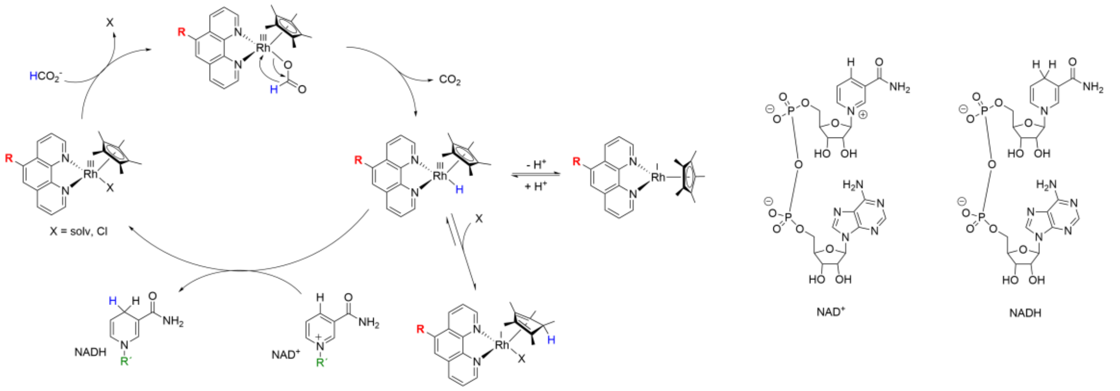

Supplementary Figure 9: Schematic representation of the mechanism of formate driven NADH formation.[11,12]

Catalysis Data

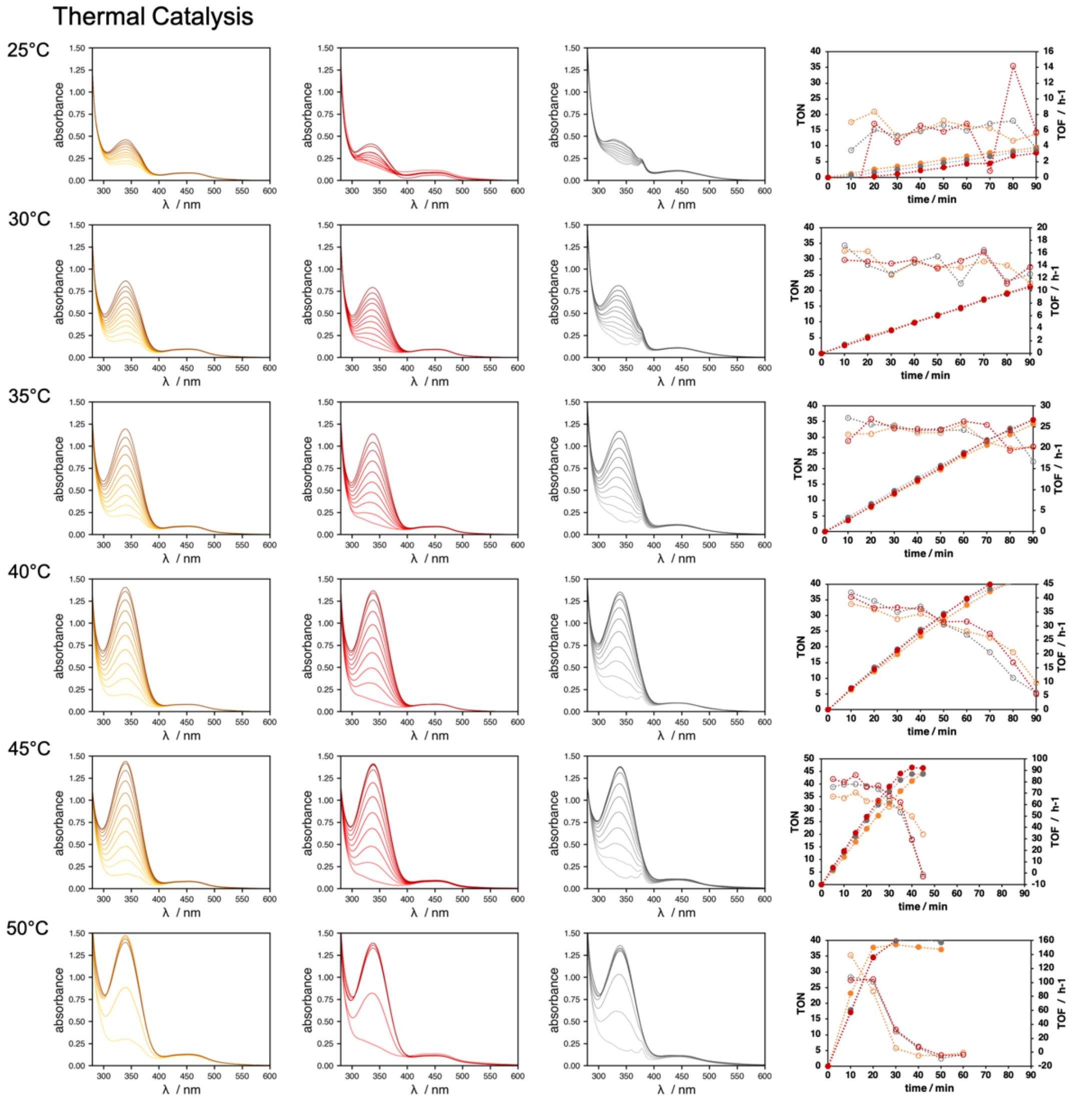

Supplementary Figure 10: Formate driven catalysis of complexes **2** (orange), **4** (red) and **5** (grey) at the respective temperatures (50 mM $NaHCO_2$, 250 µM NAD^+, 5 µM catalyst, ACN/H_2O, 1/9, v/v). Time interval per line 10 min (at 45°C: 5 min time interval).) Dotted lines added as a visual guidance in the TON-TOF-plots where the color code is in accordance with the UV/vis absorption spectra.

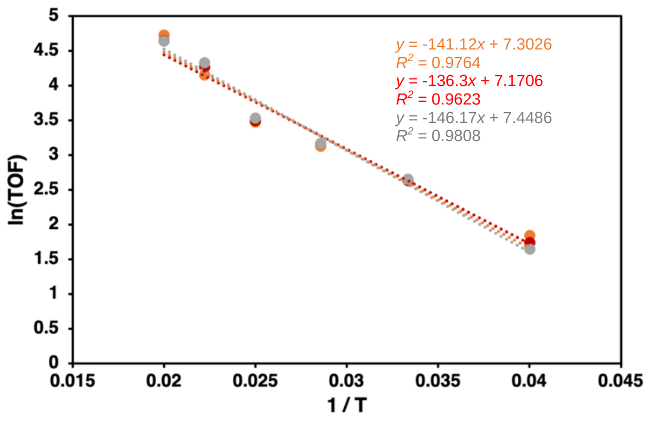

Supplementary Figure 11: Arrhenius fit over temperature dependent turnover frequency data of the formate driven catalysis. Data for complexes **2**, **4** and **5** are represented by orange, red and gray color.

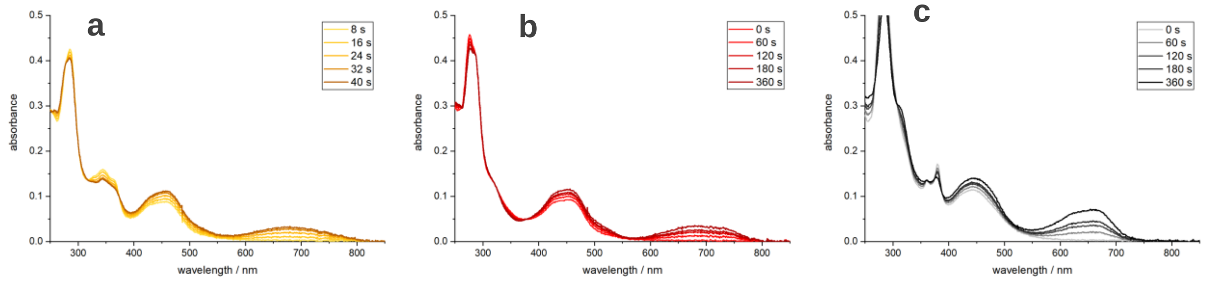

Supplementary Figure 12: Photocatalytic Rh(I) generation of complexes **2** (yellow, a), **4** (red, b) and **5** (grey, c). (0.1 M TEA, 5 µM catalyst, ACN/H_2O, 1/2 v/v).

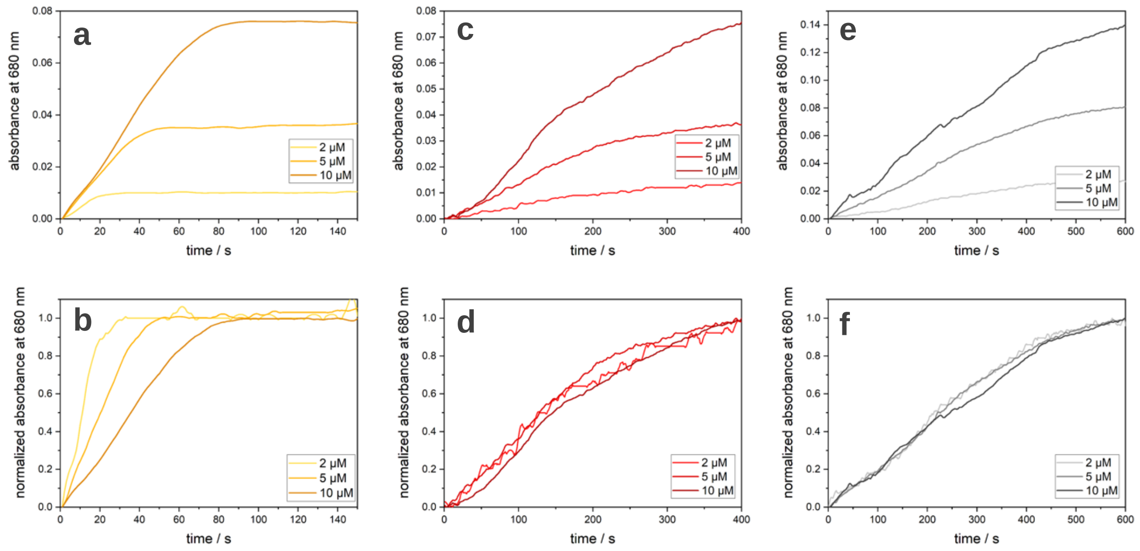

Supplementary Figure 13: Absorbance at 680 nm (a, c, e) and normalized absorbance (b, d, f) during irradiation (470 nm, 54 mW/cm^2) of deaerated photocatalyst solutions at concentrations ranging from 2 µM to 10 µM (0.12 M TEA, acetonitrile/water (1/9, v/v)). Complexes **2** (a, b), **4** (c, d) and **5** (e, f) are represented by orange, red and gray solid lines, respectively.

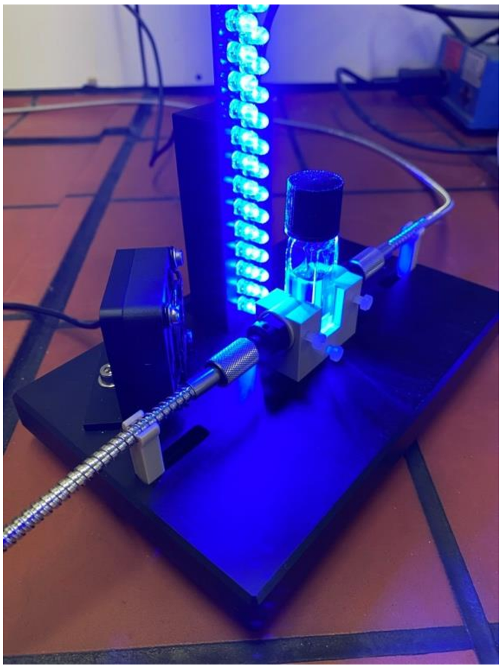

Supplementary Figure 14: Irradiation/detection setup for UV/vis spectroscopic monitoring of Rh(I) formation for concentration dependent studies.

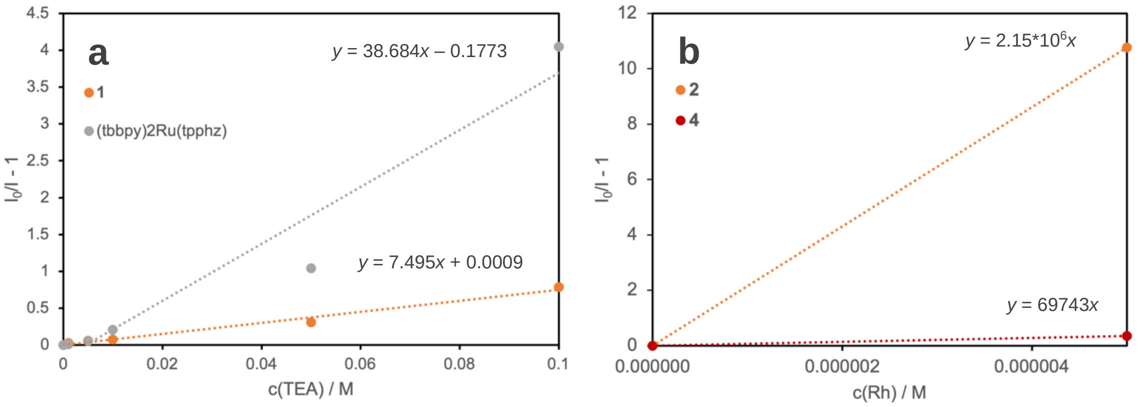

Supplementary Figure 15: All measurements were performed in de-aerated acetonitrile. a: Stern Volmer quenching of 5 µM solutions of the two mononuclear complexes **1** (orange dots and dotted line) and $[(tbbpy)_2Ru(tpphz)](PF_6)_2$ (gray dots and dotted lines). **3** was not quenched at all and was therefore omitted from the plot. b: Quenching of the mononuclear complexes **1** and **3** by equal amounts of rhodium centers in their respective dyads **2** and **4**, using a concentration of 5 µM for all compounds (i.e. the luminescence of the mononuclear and dinuclear complexes is compared). Color code: **1** and **2** are represented by orange dots and dotted lines, **3** and **4** are represented by red dots and dotted lines.

Photocatalysis

Mechanism

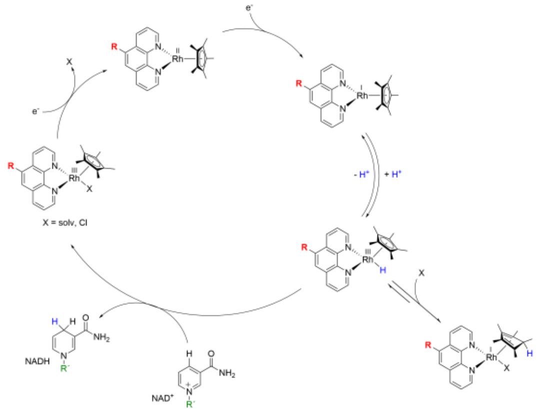

Supplementary Figure 16: Schematic representation of the mechanism during photocatalytic NADH formation.[12,13]

Catalysis Data

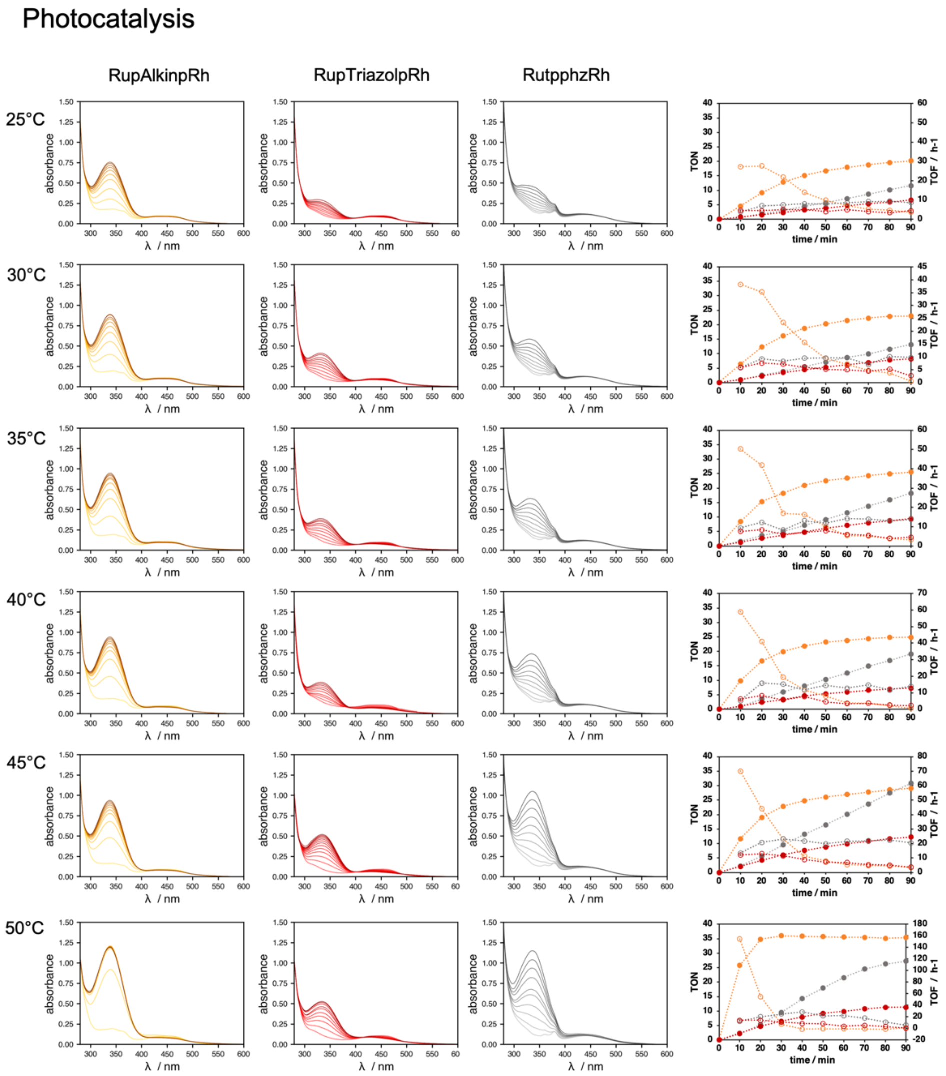

Supplementary Figure 17: Photocatalysis experiments of complexes **2** (orange), **4** (red) and **5** (grey) at the respective temperatures (0.12 M TEA, 0.1 M NaH_2PO_4, 250 µM NAD^+, 5 µM catalyst, ACN/H_2O, 1/2, v/v). Each line represents a time interval of 10 min. Dotted lines added as a visual guidance in the TON-TOF-plots where the color code is in accordance with the UV/vis absorption spectra.

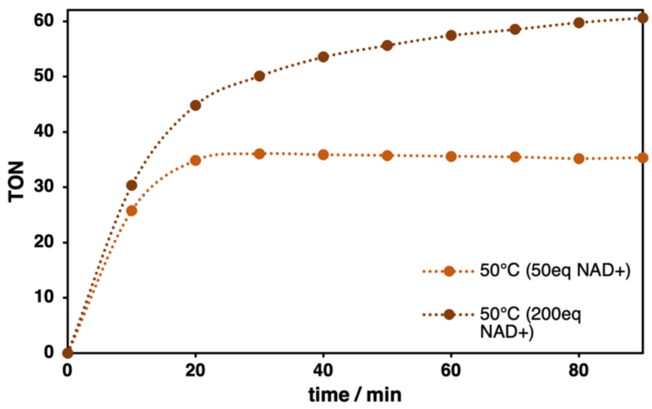

Supplementary Figure 18: Photocatalysis experiment of complex **2** at 50°C with 50 and 200 equivalents of substrate (0.12 M TEA, 0.1 M NaH_2PO_4, 250/1000 µM NAD+, 5 µM catalyst, ACN/H_2O, 1/2 v/v). Dotted lines added as a visual guidance.

Procedure for determining the selectivity of NADH formation:

For determination of the NADH selectivity of the different catalytic processes, a calibration curve using commercially available NADH (Sigma Aldrich, 97 % purity) was recorded as follows: Samples of different NADH concentrations (0 µM, 25 µM, 50 µM, 100 µM, 150 µM, 200 µM, 250 µM and 300 µM) were prepared in either ACN:H_2O = 1:9 (v:v) or ACN:H_2O = 1:2 (v:v) at air and an UV/vis absorption spectrum was recorded. Afterwards from each sample an aliquot was taken out and diluted with water to a tenth of its initial concentration using deionized water. With these samples, emission spectra were recorded using λ_{exc} = 340 nm. After considering the 97 % purity of the utilized commercially available NADH, a plot of E(340 nm) vs. emission intensity(462 nm) could be plotted that gives the expected emission intensity for a certain absorbance increase at 340 nm if during the catalytic process NADH would have been generated in 100 % selectivity. To determine the true selectivity of the catalytic process, the actually recorded emission intensity was divided by the emission intensity that would have been expected for 100 % NADH-selectivity based on the recorded absorbance increase at 340 nm.

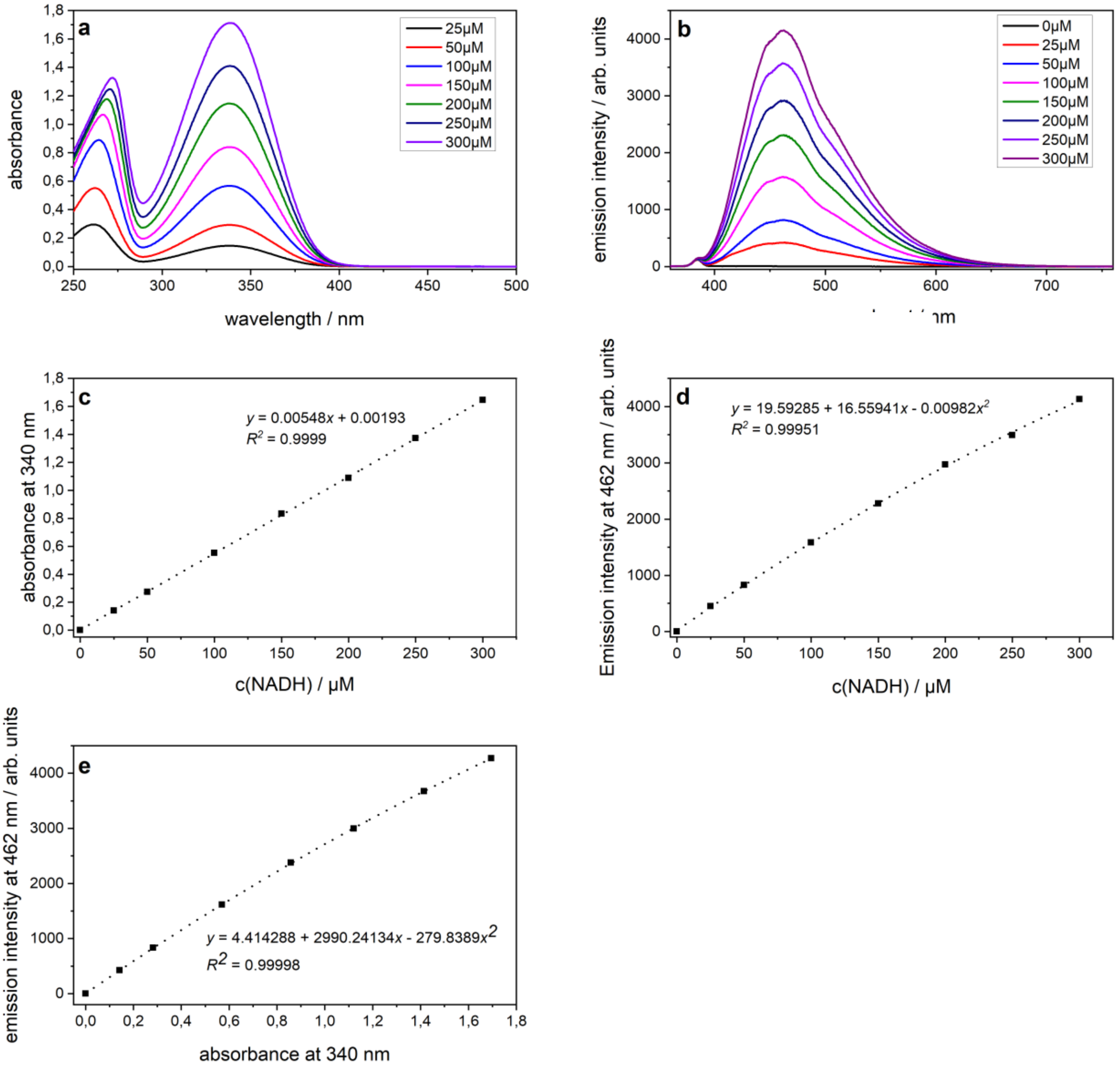

Supplementary Figure 19: UV/vis absorption (a,c) und emission (b,d) data utilized in ACN:H_2O = 1:9 (v:v) to determine the expectation curve for 100% NADH formation selectivity (e). See text above for further details.

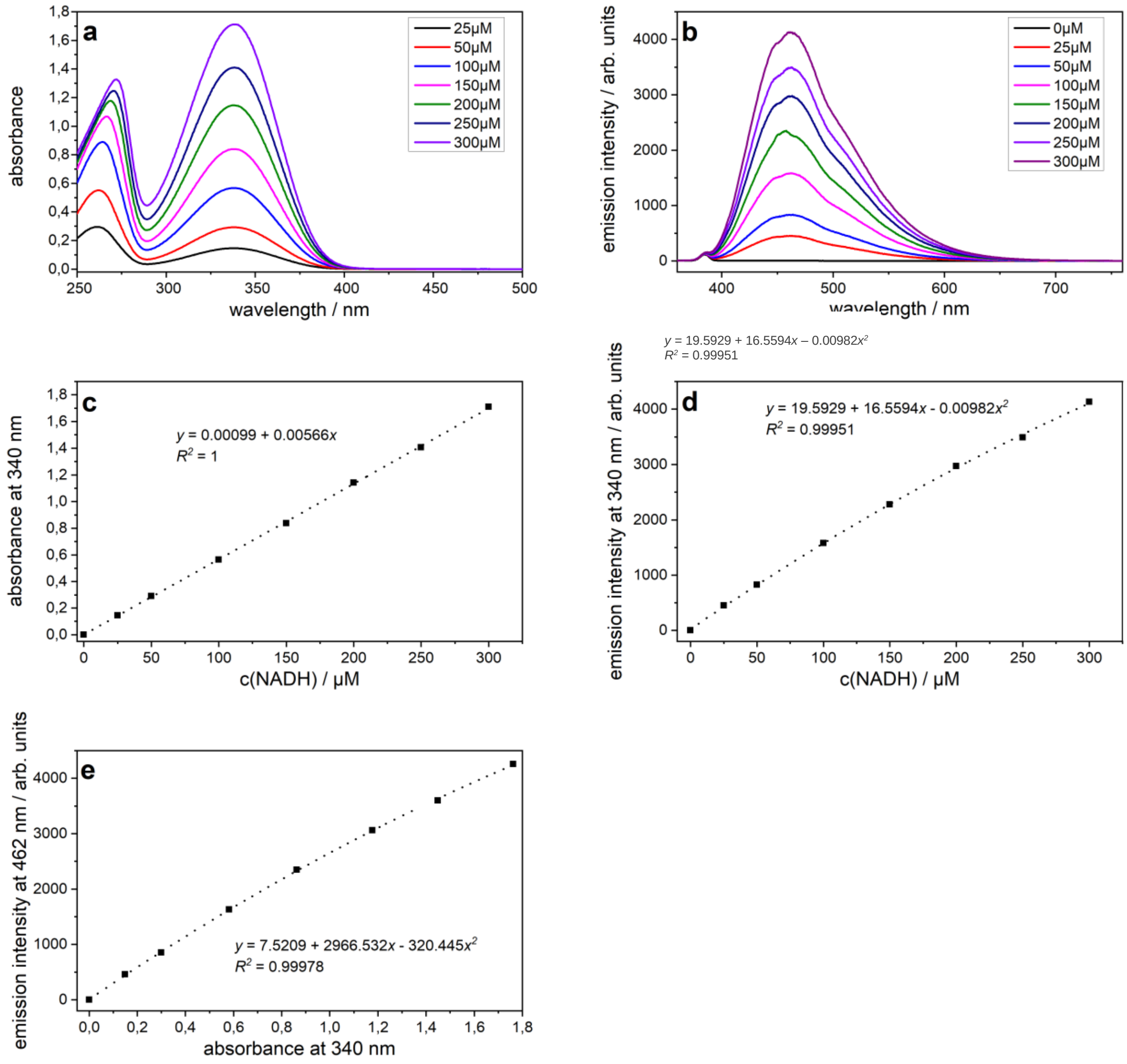

Supplementary Figure 20: UV/vis absorption (a,c) und emission (b,d) data utilized in ACN:H_2O = 1:2 (v:v) to determine the expectation curve for 100% NADH formation selectivity (e). See text above for further details.

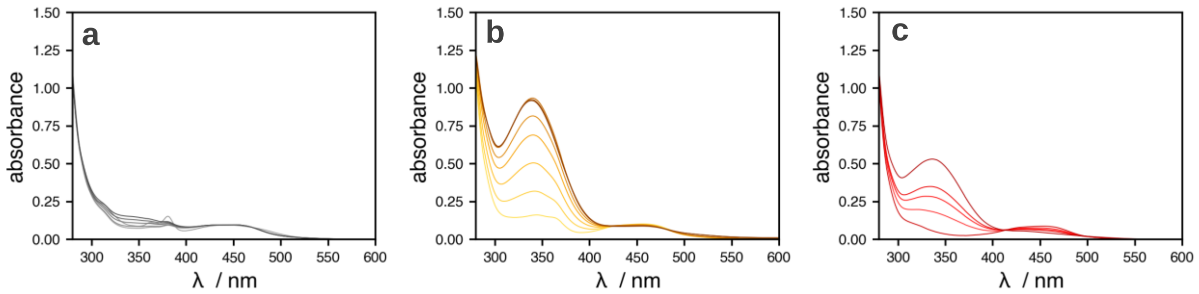

Supplementary Figure 21: Irradiation of [(tbbpy)$_2$Rutpphz](PF_6)$_2$ (grey, a), **1** (orange, b), and **3** (red, c) under photocatalytic conditions. (45°C, 0.12 M TEA, 0.1 M NaH_2PO_4, 250 µM NAD^+, 5 µM catalyst, ACN/H_2O, 1/2 v/v).

Supplementary Table 3: Comparison of NADH formation selectivity under photocatalytic conditions between complexes with (**2**,**4**,**5**) and without (**1**,**3**, [(tbbpy)$_2$Ru(tpphz)](PF$_6$)$_2$) [Rh(Cp*)Cl catalytic center (45°C, 0.12 M TEA, 0.1 M NaH$_2$PO$_4$, 250 µM NAD$^+$, 5 µM catalyst, ACN/H$_2$O, 1/2, v/v).

	Selectivity
**2**	90%
**4**	98%
**5**	96%

	Selectivity
**1**	1.2%
**3**	7.1%
[(tbbpy)$_2$Ru(tpphz)](PF$_6$)$_2$	32%

Investigations regarding the deactivation of catalyst 2:

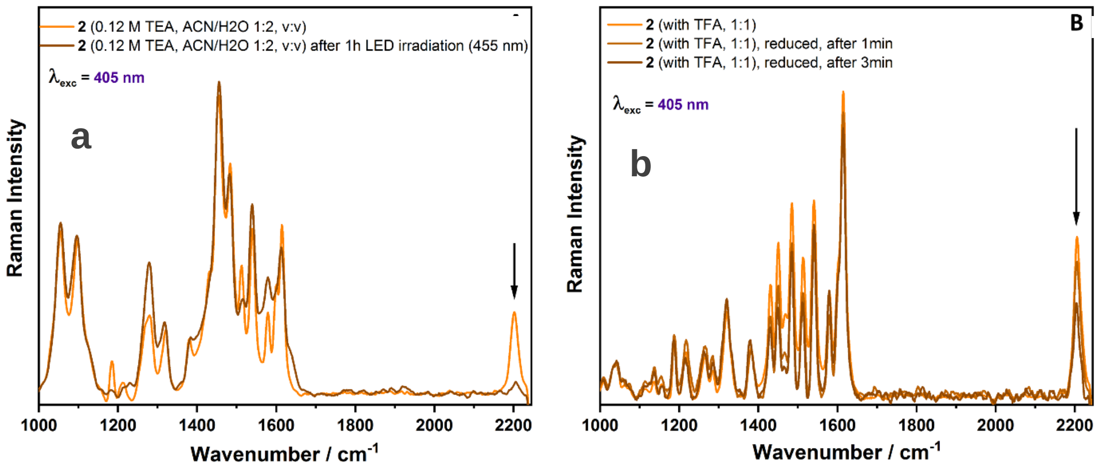

Supplementary Figure 22: a: RR spectra of deaerated photocatalyst solutions of **2** (0.12 M TEA, ACN/H2O, 1/2, v/v) upon excitation at 405 nm before (orange spectrum) and after (brown spectrum) irradiation (455 nm). b: RR spectra of de-aerated photocatalyst solutions of **2** (TFA, ACN) upon excitation at 405 nm before (orange spectrum) and after electrochemical reduction (brown spectra) of the catalytic center.

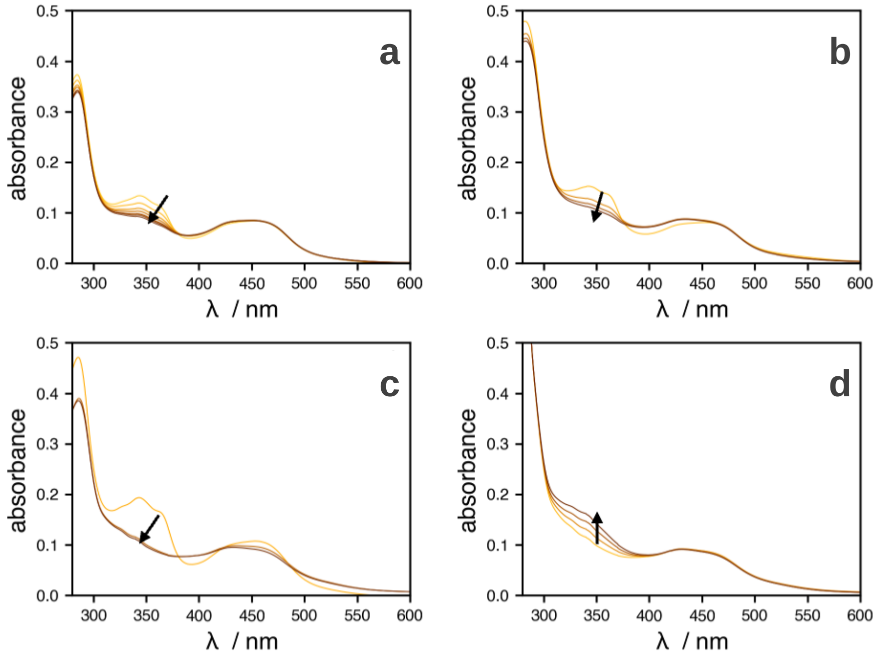

Supplementary Figure 23: a: Degradation of the π-π* band of complex **2** (associated with loss of alkyne functionality) under formate driven catalysis conditions without substrate (45 °C, 50 mM $NaHCO_2$, 5 µM catalyst, ACN/H_2O, 1/9, v/v, each line 10 min). b: Degradation of the π-π* band of complex **1** during formate driven catalysis conditions without substrate in presence of [(phen)RhCp*Cl]Cl as model catalyst (45 °C, 50 mM $NaHCO_2$, 5 µM catalyst, 5µM **1**, ACN/H_2O, 1/9, v/v, each line 10 min). c: Light driven degradation of the π-π* band of complex **2** during irradiation under photocatalysis conditions without substrate. (45°C, 0.12 M TEA, 0.1 M NaH_2PO_4, 5 µM catalyst, ACN/H_2O, 1/2 v/v, each line 15 min). d: Photocatalysis after light driven degradation (see c) by addition of NAD^+ to the same solution (250 µM NAD^+, each line 10 min). The arrows indicate the direction in which the relevant bands develop over time.

Resonance Raman spectra

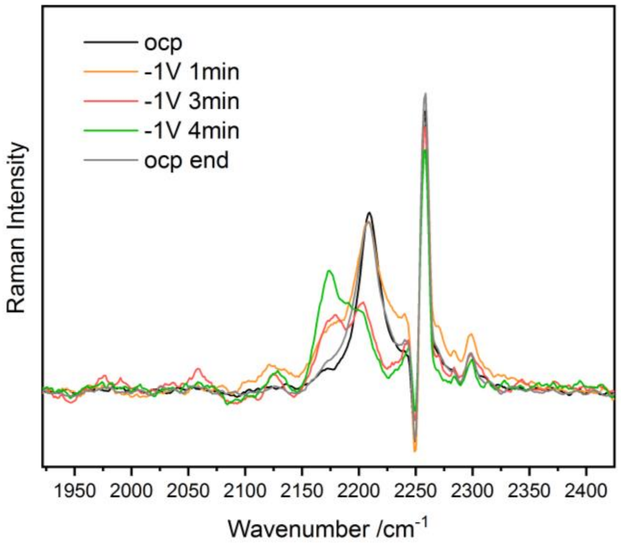

Supplementary Figure 24: Experimental rR spectra of non-reduced (ocp) and electrochemically doubly reduced (-1 V) complex **1**, excited at 405 nm (grating 2400 / mm). The vibrational mode associated with the -C≡C- triple bond at 2212 cm^{-1} is shifted to lower wavenumbers upon reduction, indicating an extension of the chromophoric system in complex **1** by the alkynyl functionality. The sharp negative peak at 2250 cm^{-1} results from subtracting the solvent spectrum of ACN.

Transient absorption spectra

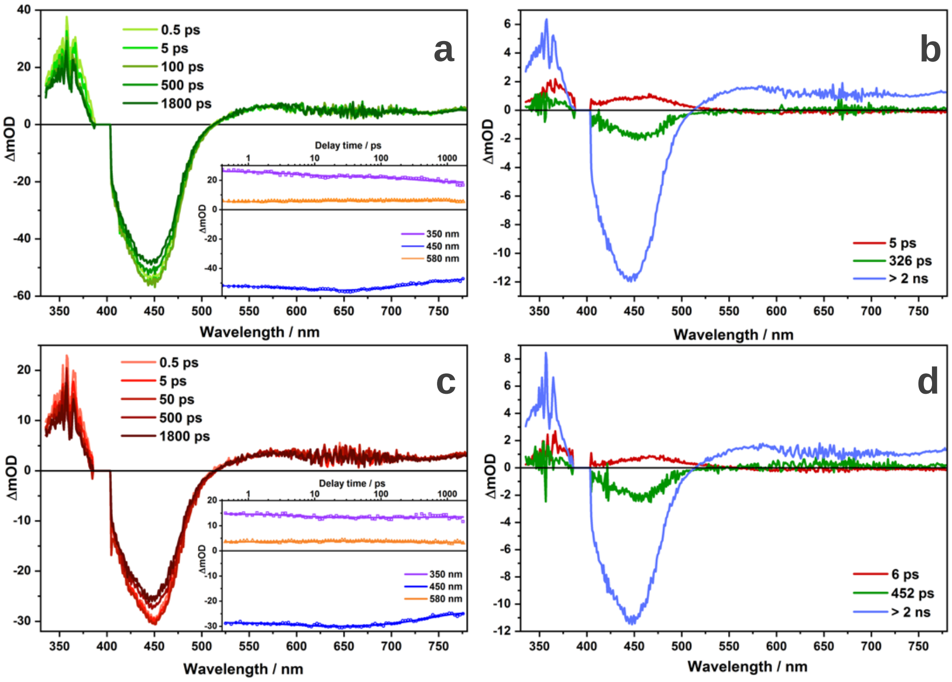

Supplementary Figure 25: Transient absorption spectra recorded for **3** (a) and **4** (c) in ACN upon pumping at 400 nm at different delay times. Kinetic traces of **3** (Inset in a) and **4** (Inset in c) at selected wavelengths. Decay-associated spectra and corresponding time constants of **3** (b) **4** (d) derived from a global multiexponential fit applied on the transient absorption data.

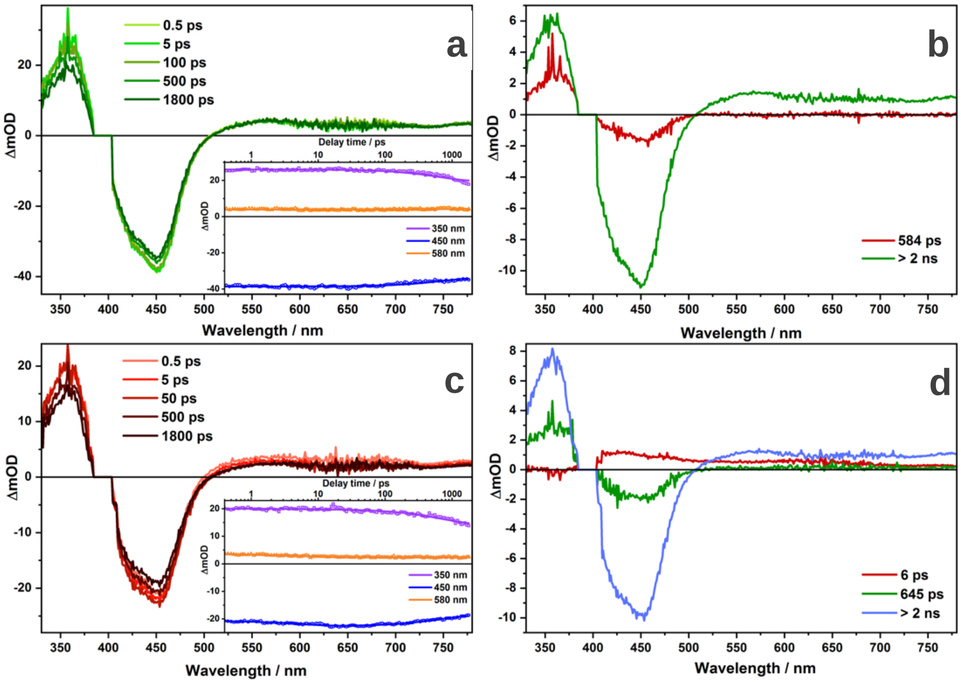

Supplementary Figure 26: Transient absorption spectra recorded for **3** (a) and **4** (c) in dichloromethane (DCM) upon pumping at 400 nm at different delay times. Kinetic traces of **3** (Inset in a) and **4** (Inset in c) at selected wavelengths. Decay-associated spectra and corresponding time constants of **3** (b) and **4** (d) derived from a global multiexponential fit applied on the transient absorption data.

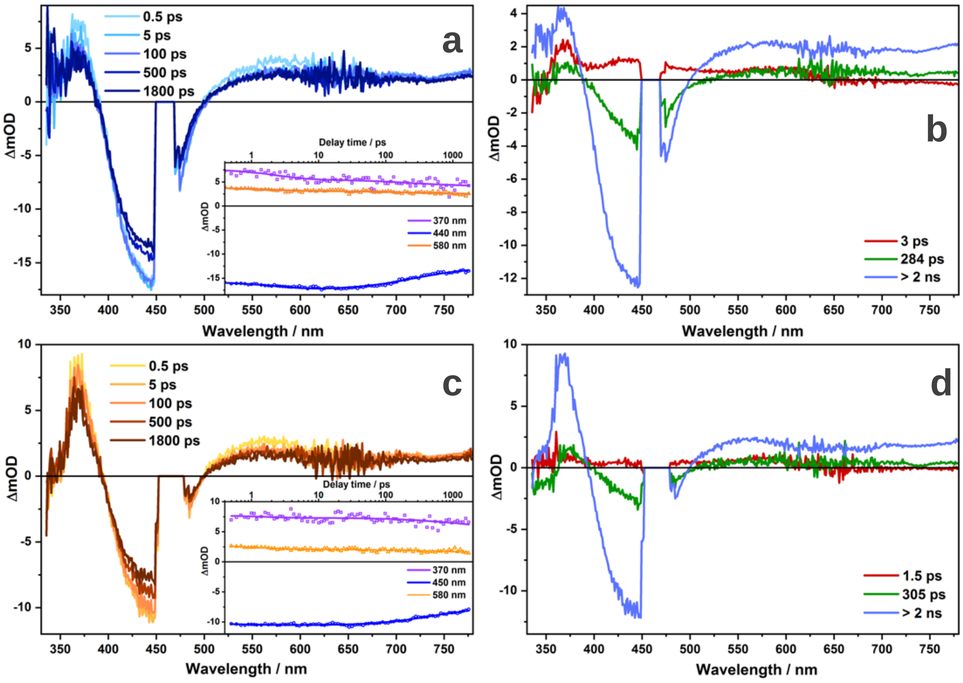

Supplementary Figure 27: Transient absorption spectra recorded for **1** (a) and **2** (c) in ACN upon pumping at 470 nm at different delay times. Kinetic traces of **1** (Inset in a) and **2** (Inset in c) at selected wavelengths. Decay-associated spectra and corresponding time constants of **1** (b) and **2** (d) derived from a global multiexponential fit applied on the transient absorption data.

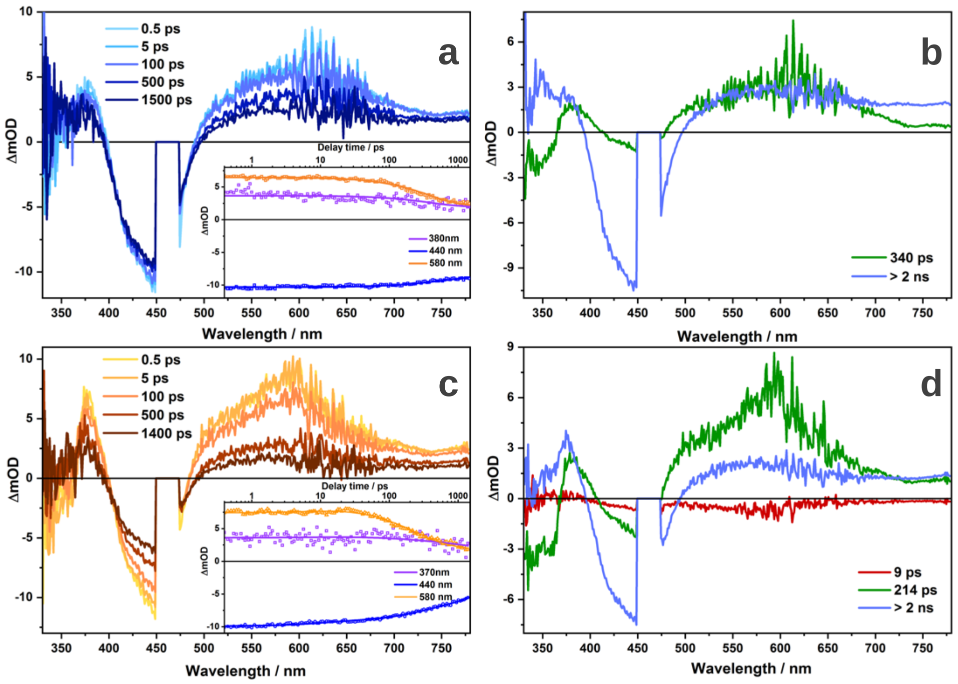

Supplementary Figure 28: Transient absorption spectra recorded for **1** (a) and **2** (c) in dichloromethane (DCM) upon pumping at 470 nm at different delay times. Kinetic traces of **1** (Inset in a) and **2** (Inset in c) at selected wavelengths. Decay-associated spectra and corresponding time constants of **1** (b) and **2** (d) derived from a global multiexponential fit applied on the transient absorption data.

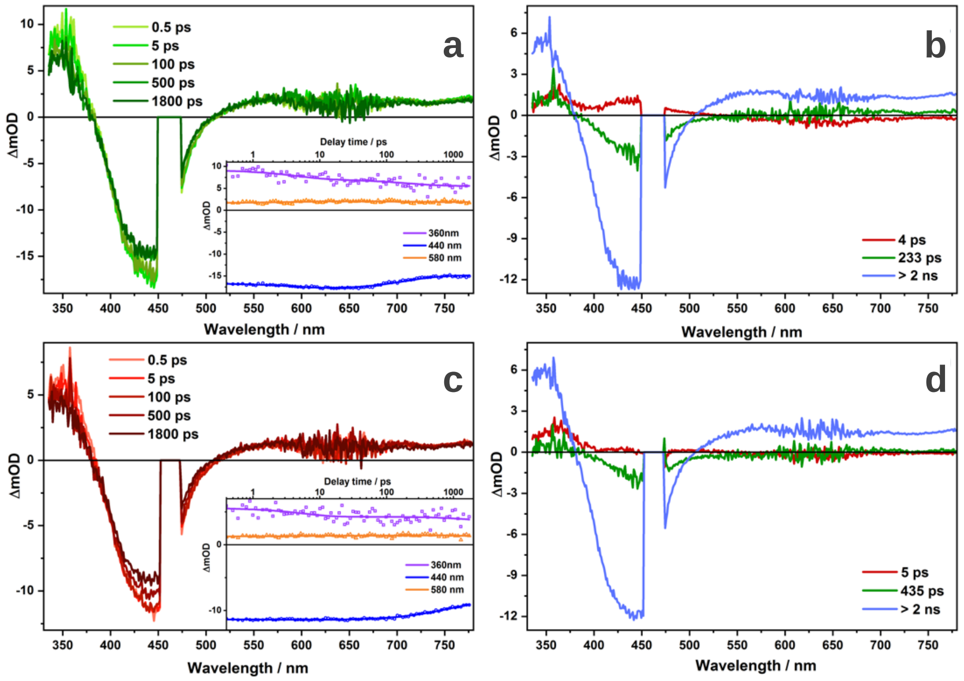

Supplementary Figure 29: Transient absorption spectra recorded for **3** (a) and **4** (c) in ACN upon pumping at 470 nm at different delay times. Kinetic traces of **3** (Inset in a) and **4** (Inset in c) at selected wavelengths. Decay-associated spectra and corresponding time constants of **3** (b) and **4** (d) derived from a global multiexponential fit applied on the transient absorption data.

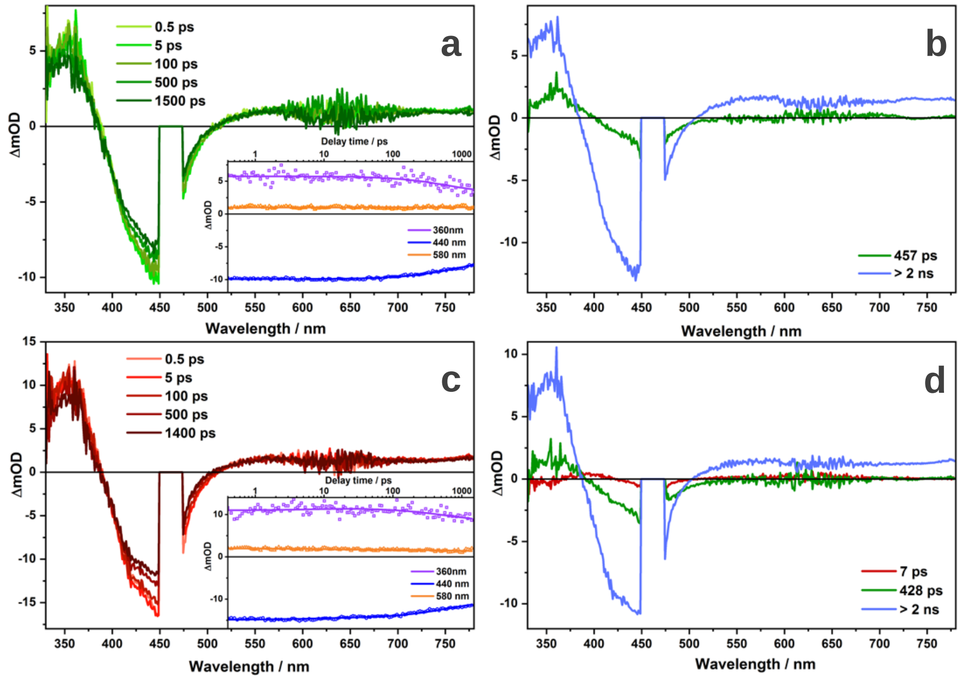

Supplementary Figure 30: Transient absorption spectra recorded for **3** (a) and **4** (c) in dichloromethane (DCM) upon pumping at 470 nm at different delay times. Kinetic traces of **3** (Inset in a) and **4** (Inset in c) at selected wavelengths. Decay-associated spectra and corresponding time constants of **3** (b) and **4** (d) derived from a global multiexponential fit applied on the transient absorption data.

Supplementary Table 4: Transient absorption time constants derived by global fit of the femtosecond time-resolved absorption data excited at 400 nm for **1**-**4** in acetonitrile (ACN) and dichloromethane (DCM). For all fits there is a third time constant larger than 2 ns (see Figure 9, 10, S21-S26).

Compound	Solvent	τ_1 / ps	τ_2 / ps
**1**	ACN	3	272
**1**	DCM	-	255
**2**	ACN	3	562
**2**	DCM	9	222
**3**	ACN	5	326
**3**	DCM	-	584
**4**	ACN	6	452
**4**	DCM	6	645

Supplementary Table 5: Transient absorption time constants derived by global fit of the femtosecond time-resolved absorption data excited at 470 nm for **1**-**4** in acetonitrile (ACN) and dichloromethane (DCM). For all fits there is a third time constant larger than 2 ns (see Figure 9, 10, S21-26).

Compound	Solvent	τ_1 / ps	τ_2 / ps
**1**	ACN	3	284
**1**	DCM	-	340
**2**	ACN	2	305
**2**	DCM	9	214
**3**	ACN	4	233
**3**	DCM	-	340
**4**	ACN	5	435
**4**	DCM	7	428

Supplementary References

1. Siebert, R. *et al.* Spectroscopic investigation of the ultrafast photoinduced dynamics in π-conjugated terpyridines. *ChemPhysChem* **10**, 910–919 (2009).
2. Karnahl, M. *et al.* Tuning of photocatalytic hydrogen production and photoinduced intramolecular electron transfer rates by regioselective bridging ligand substitution. *ChemPhysChem* **12**, 2101–2109 (2011).
3. Dobryakov, A. L., Kovalenko, S. A. & Ernsting, N. P. Coherent and sequential contributions to femtosecond transient absorption spectra of a rhodamine dye in solution. *Journal of Chemical Physics* **123**, 044502 (2005).
4. Müller, C., Pascher, T., Eriksson, A. & Uhlig, J. KiMoPack. (2022) doi:10.5281/zenodo.5720587.
5. Barthelmes, K. *et al.* New ruthenium Bis(terpyridine) methanofullerene and pyrrolidinofullerene complexes: Synthesis and electrochemical and photophysical properties. *Inorganic Chemistry* **54**, 3159–3171 (2015).
6. Stumper, A. *et al.* Efficient Access to 5-Bromo- and 5,6-Dibromophenanthroline Ligands. *European Journal of Inorganic Chemistry* **2017**, 3799–3810 (2017).
7. Wintergerst, P., Mengele, A. K., Nauroozi, D., Tschierlei, S. & Rau, S. Impact of Alkyne Functionalization on Photophysical and Electrochemical Properties of 1,10-Phenanthrolines and Their Ru II Complexes. *European Journal of Inorganic Chemistry* **2019**, 1988–1992 (2019).
8. Ohata, J. *et al.* Luminogenic iridium azide complexes. *Chemical Communications* **51**, 15192–15195 (2015).
9. Mengele, A. K., Seibold, G. M., Eikmanns, B. J. & Rau, S. Coupling Molecular Photocatalysis to Enzymatic Conversion. *ChemCatChem* **9**, 4369–4376 (2017).
10. Mengele, A. K., Kaufhold, S., Streb, C. & Rau, S. Generation of a stable supramolecular hydrogen evolving photocatalyst by alteration of the catalytic center. *Dalton Transactions* **45**, 6612–6618 (2016).
11. Pitman, C. L., Finster, O. N. L. & Miller, A. J. M. Cyclopentadiene-mediated hydride transfer from rhodium complexes. *Chemical Communications* **52**, 9105–9108 (2016).
12. Marrone, A. & Fish, R. H. DFT Mechanism Studies: Biomimetic 1,4-NADH Chemoselective, Co-factor Regeneration with [Cp*Rh(bpy)H]+, in Tandem with the Biocatalysis Pathways of a Core Model of the (HLADH)-Zn(II) Mediated Enzyme, in the Enantioselective Reduction of Achiral Ketones to Chiral S-Alcohols. *Journal of Organometallic Chemistry* **943**, 121810 (2021).
13. Zedler, L. *et al.* Unraveling the Light-Activated Reaction Mechanism in a Catalytically Competent Key Intermediate of a Multifunctional Molecular Catalyst for Artificial Photosynthesis. *Angewandte Chemie - International Edition* **58**, 13140–13148 (2019).
