# Supplementary material for: Outpacing conventional nicotinamide hydrogenation catalysis by a strongly communicating heterodinuclear photocatalyst
Source: Nat Commun. 2022 May 9;13:2538. doi: 10.1038/s41467-022-30147-4 (PMC9085789; doi:10.1038/s41467-022-30147-4)
Supplement: Supplementary file 3 — Reporting Summary [file 41467_2022_30147_MOESM3_ESM.pdf]

nature portfolio

Corresponding author(s): Benjamin Dietzek-Ivanšić
Sven Rau

Last updated by author(s): Mar 8, 2022

Reporting Summary

Nature Portfolio wishes to improve the reproducibility of the work that we publish. This form provides structure for consistency and transparency in reporting. For further information on Nature Portfolio policies, see our Editorial Policies and the Editorial Policy Checklist.

Statistics

For all statistical analyses, confirm that the following items are present in the figure legend, table legend, main text, or Methods section.

n/a	Confirmed	
x		The exact sample size (*n*) for each experimental group/condition, given as a discrete number and unit of measurement
	x	A statement on whether measurements were taken from distinct samples or whether the same sample was measured repeatedly
x		The statistical test(s) used AND whether they are one- or two-sided<br>*Only common tests should be described solely by name; describe more complex techniques in the Methods section.*
x		A description of all covariates tested
x		A description of any assumptions or corrections, such as tests of normality and adjustment for multiple comparisons
x		A full description of the statistical parameters including central tendency (e.g. means) or other basic estimates (e.g. regression coefficient) AND variation (e.g. standard deviation) or associated estimates of uncertainty (e.g. confidence intervals)
x		For null hypothesis testing, the test statistic (e.g. *F*, *t*, *r*) with confidence intervals, effect sizes, degrees of freedom and *P* value noted<br>*Give P values as exact values whenever suitable.*
x		For Bayesian analysis, information on the choice of priors and Markov chain Monte Carlo settings
x		For hierarchical and complex designs, identification of the appropriate level for tests and full reporting of outcomes
x		Estimates of effect sizes (e.g. Cohen's *d*, Pearson's *r*), indicating how they were calculated

Our web collection on statistics for biologists contains articles on many of the points above.

Software and code

Policy information about availability of computer code

Data collection	Femtosecond transient absorption data: FS software version 6.4.47.11, Pascher Instruments AB, Sweden,<br>Nanosecond transient absorption and time resolved emission data: NS software version 16.4.2, Pascher Instruments AB, Sweden,<br>Resonance Raman spectroscopy: WinSpec32 version 2.5.20.2, Princeton Instruments, USA<br>UV/vis absorption spectroscopy: Spectra Manager Version 2, Version 2.02.12, JASCO Corporation, Japan.<br>Emission spectroscopy: Spectra Manager Version 2, Version 2.07.00, JASCO Corporation, Japan.<br>NMR spectroscopy: Bruker Icon NMR 5.2.4 Build 12.6, for TopSpin 4.1.4, Bruker BioSpin 2021.<br>Electrochemistry: CHI Version 17.02, CH Instruments Inc., Austin, TX, USA
Data analysis	Femtosecond transient absorption data: python software tool KiMoPack (DOI:10.5281/zenodo.5720587) and OriginPro 2020b 64-bit version 9.7.5.184, Originlab, USA<br>Nanosecond transient absorption and time resolved emission data: NS software version 4.2.3, Pascher Instruments AB, Sweden and OriginPro 2020b 64-bit version 9.7.5.184, Originlab, USA<br>Resonance Raman spectroscopy: OriginPro 2020b 64-bit version 9.7.5.184, Originlab, USA<br>NMR spectroscopy: MestReNova 14.2.0, Mestrelab Research S.L.<br>Catalysis Data: Microsoft Excel, Version 2108.<br>Electochemistry: Microsoft Excel, Version 2108.<br>Mass spectrometry: Compass DataAnalysisViewer Version 4.2 (Build 395), Bruker Daltonik GmbH, Germany.

For manuscripts utilizing custom algorithms or software that are central to the research but not yet described in published literature, software must be made available to editors and reviewers. We strongly encourage code deposition in a community repository (e.g. GitHub). See the Nature Portfolio guidelines for submitting code & software for further information.

Data

Policy information about availability of data

All manuscripts must include a data availability statement. This statement should provide the following information, where applicable:

- Accession codes, unique identifiers, or web links for publicly available datasets
- A description of any restrictions on data availability
- For clinical datasets or third party data, please ensure that the statement adheres to our policy

The datasets generated and analysed during the current study are available from the corresponding author on reasonable request.

Field-specific reporting

Please select the one below that is the best fit for your research. If you are not sure, read the appropriate sections before making your selection.

[x] Life sciences [ ] Behavioural & social sciences [ ] Ecological, evolutionary & environmental sciences

For a reference copy of the document with all sections, see nature.com/documents/nr-reporting-summary-flat.pdf

Life sciences study design

All studies must disclose on these points even when the disclosure is negative.

Sample size	Not applicable. Solutions of the substances have been prepared and measured.
Data exclusions	Spectroscopy: sample integrity was tested before and after measurement by UV-Vis spectroscopy, data sets affected by sample degradation during measurements have been excluded
Replication	spectroscopic data: reproducibility was tested as described, the data shown is one representative measurement out of two or more; catalysis data: the number of individual catalytic runs (always using a fresh solution of catalyst) was always two or more and is additionally given in every relevant figure caption.
Randomization	not applicable
Blinding	not applicable

Reporting for specific materials, systems and methods

We require information from authors about some types of materials, experimental systems and methods used in many studies. Here, indicate whether each material, system or method listed is relevant to your study. If you are not sure if a list item applies to your research, read the appropriate section before selecting a response.

Materials & experimental systems

n/a	Involved in the study	
[x]	[ ]	Antibodies
[x]	[ ]	Eukaryotic cell lines
[x]	[ ]	Palaeontology and archaeology
[x]	[ ]	Animals and other organisms
[x]	[ ]	Human research participants
[x]	[ ]	Clinical data
[x]	[ ]	Dual use research of concern

Methods

n/a	Involved in the study	
[x]	[ ]	ChIP-seq
[x]	[ ]	Flow cytometry
[x]	[ ]	MRI-based neuroimaging
