# Supplementary figures and images for: Outpacing conventional nicotinamide hydrogenation catalysis by a strongly communicating heterodinuclear photocatalyst
Source: Nat Commun. 2022 May 9;13:2538. doi: 10.1038/s41467-022-30147-4 (PMC9085789; doi:10.1038/s41467-022-30147-4)

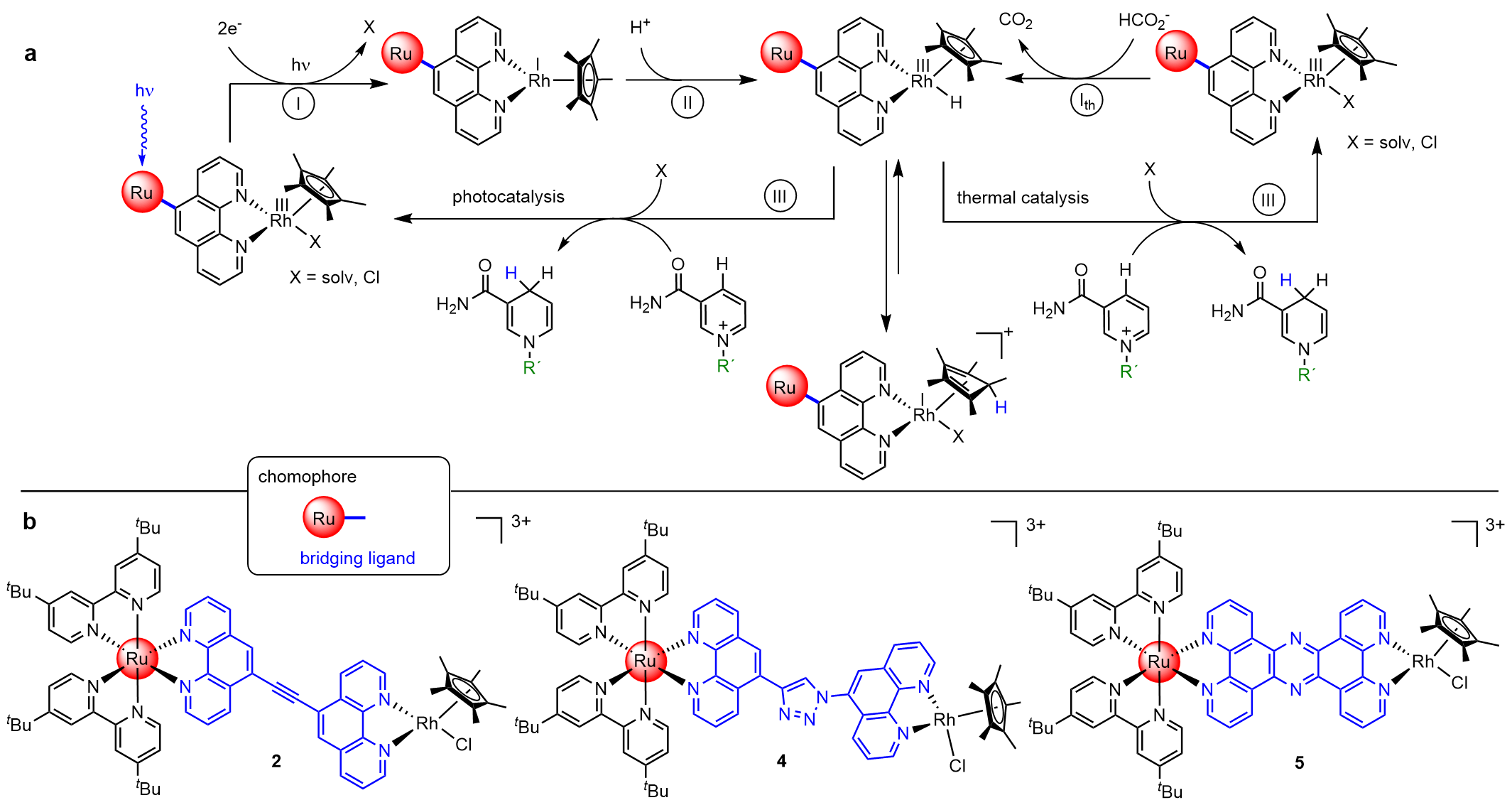

Supplement: Supplementary file 4 — Source Data [file 41467_2022_30147_MOESM4_ESM.zip › SourceData/Source Data Fig. 1/Figure1.tif]

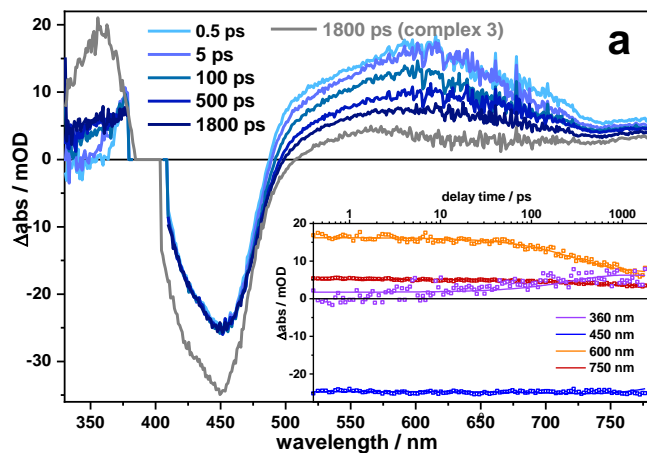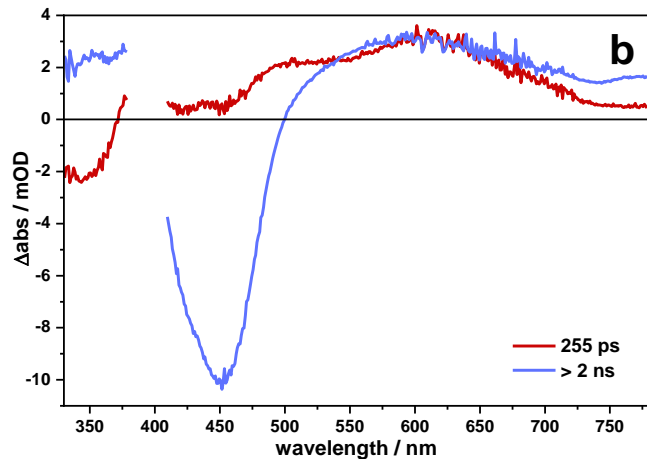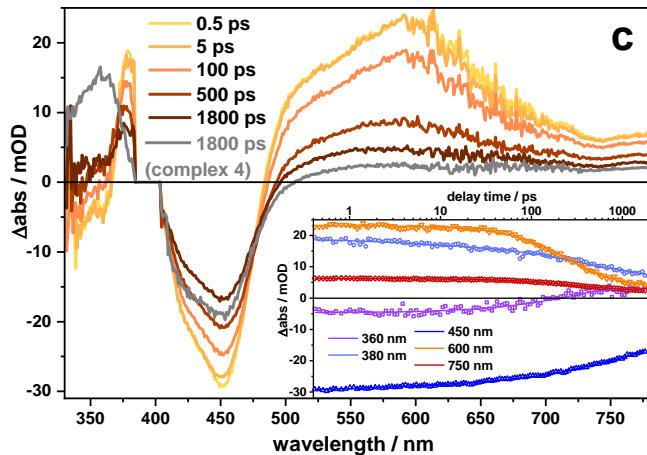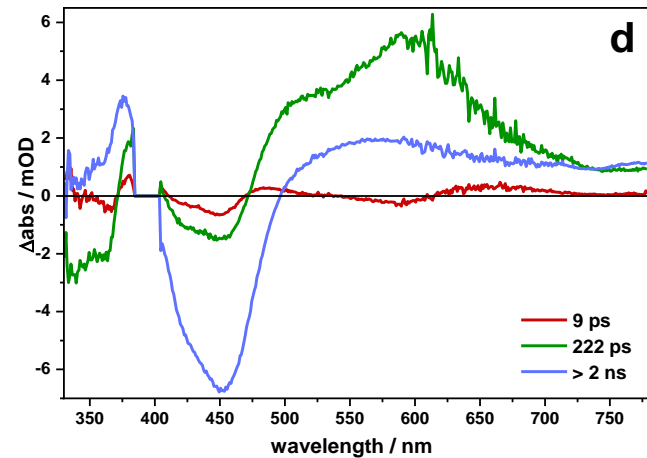

Supplement: Supplementary file 4 — Source Data [file 41467_2022_30147_MOESM4_ESM.zip › SourceData/Source Data Fig. 10/figure_10.pdf]

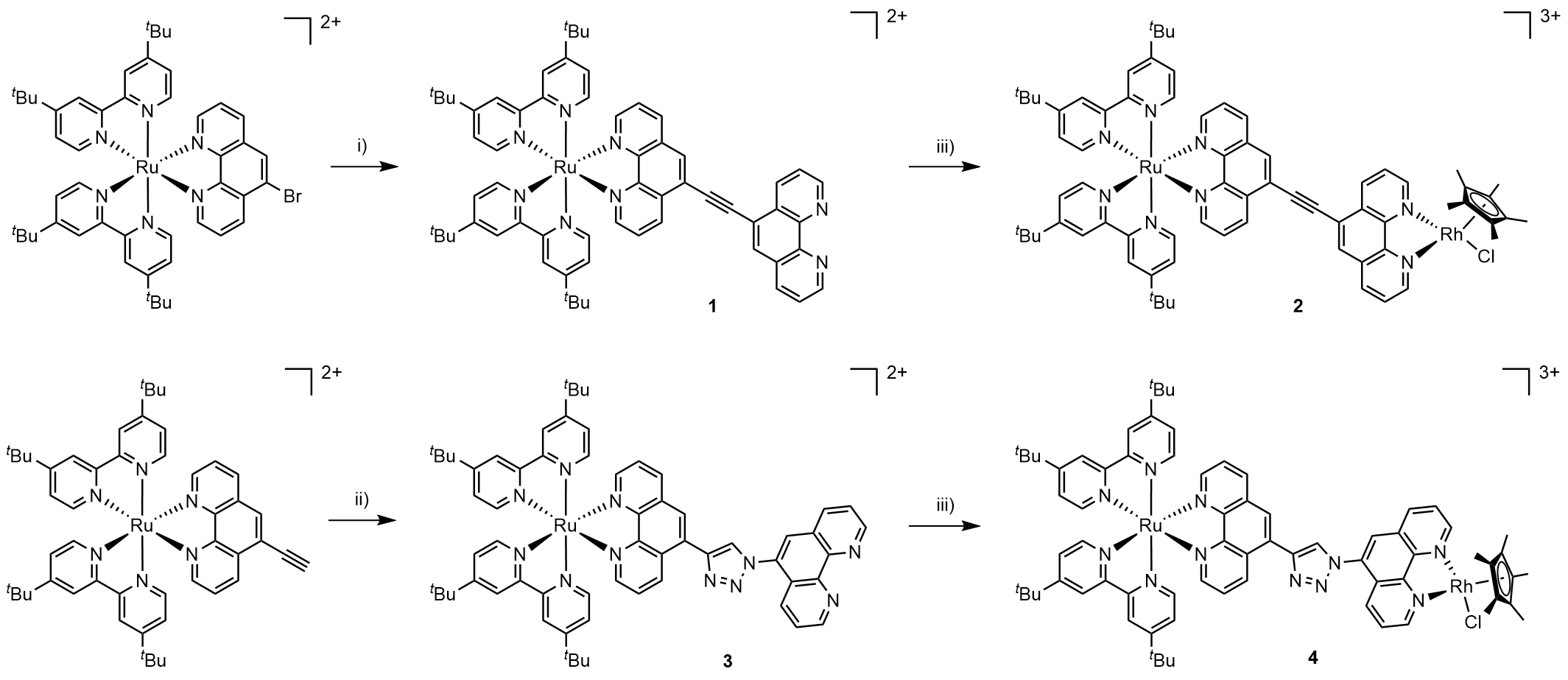

Supplement: Supplementary file 4 — Source Data [file 41467_2022_30147_MOESM4_ESM.zip › SourceData/Source Data Fig. 2/Figure_2.tif]

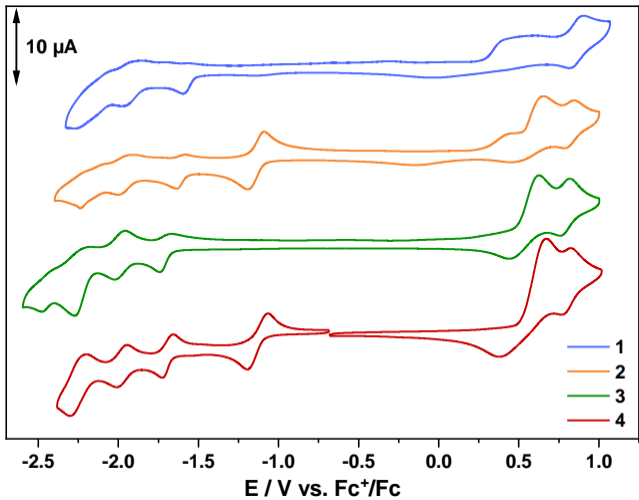

Supplement: Supplementary file 4 — Source Data [file 41467_2022_30147_MOESM4_ESM.zip › SourceData/Source Data Fig. 3/figure_3.pdf]

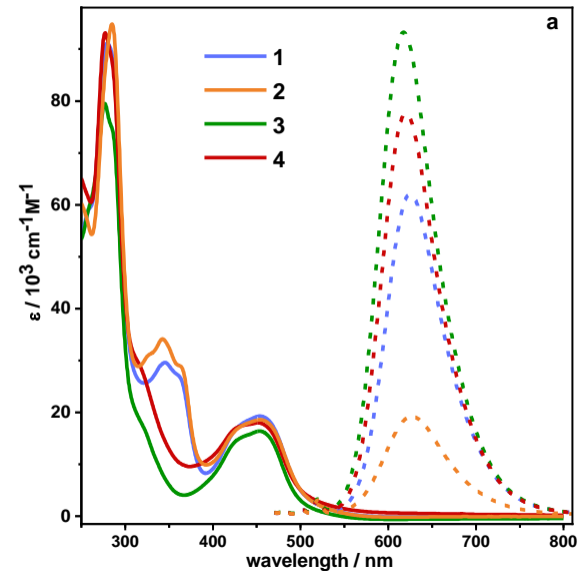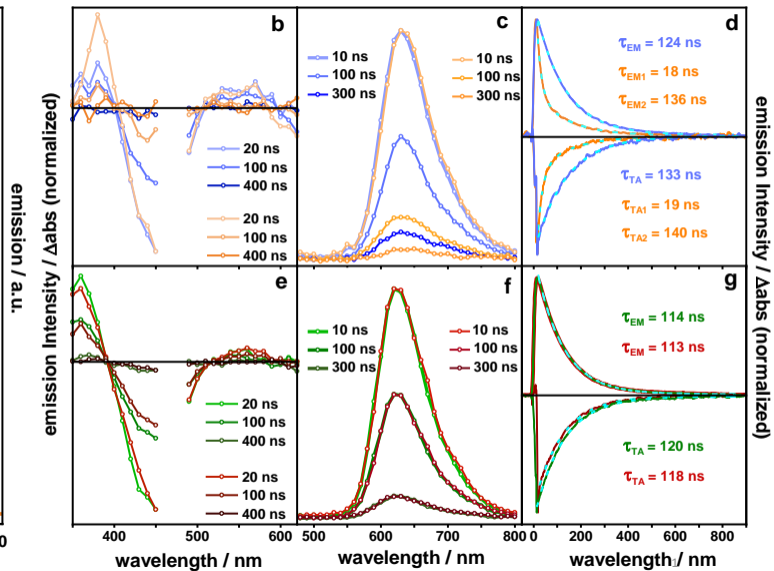

Supplement: Supplementary file 4 — Source Data [file 41467_2022_30147_MOESM4_ESM.zip › SourceData/Source Data Fig. 4/figure_4.pdf]

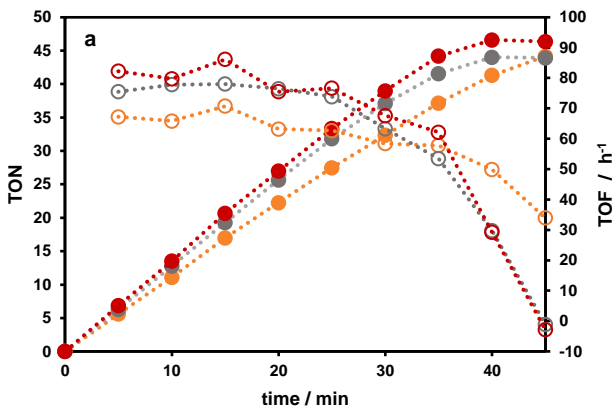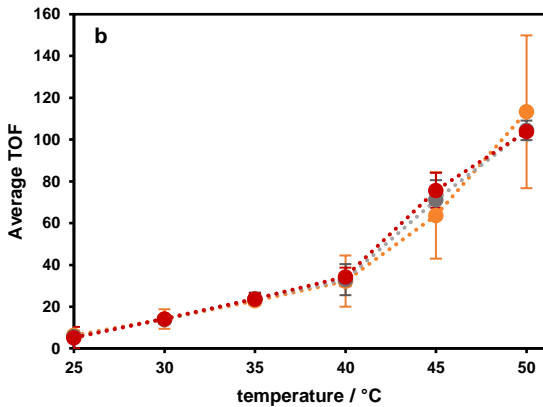

Supplement: Supplementary file 4 — Source Data [file 41467_2022_30147_MOESM4_ESM.zip › SourceData/Source Data Fig. 5/figure_5.pdf]

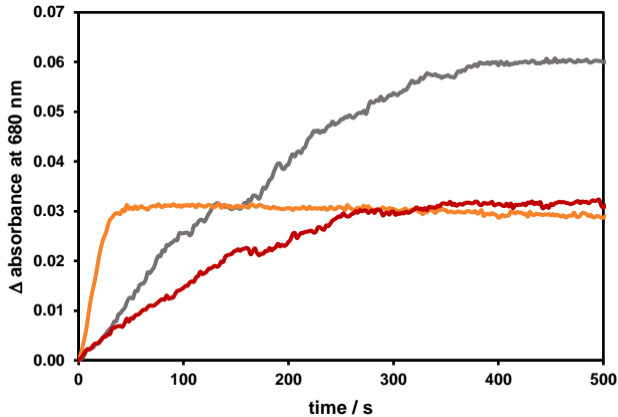

Supplement: Supplementary file 4 — Source Data [file 41467_2022_30147_MOESM4_ESM.zip › SourceData/Source Data Fig. 6/Figure_6.pdf]

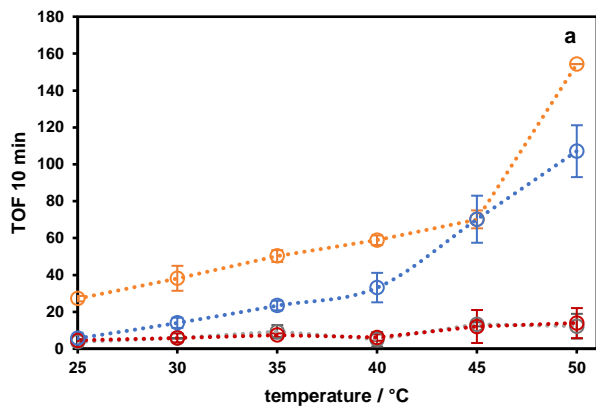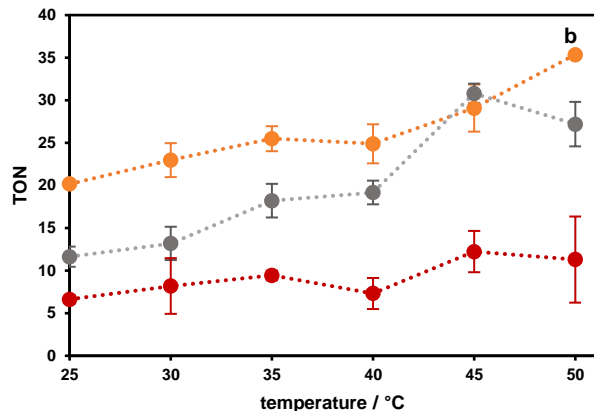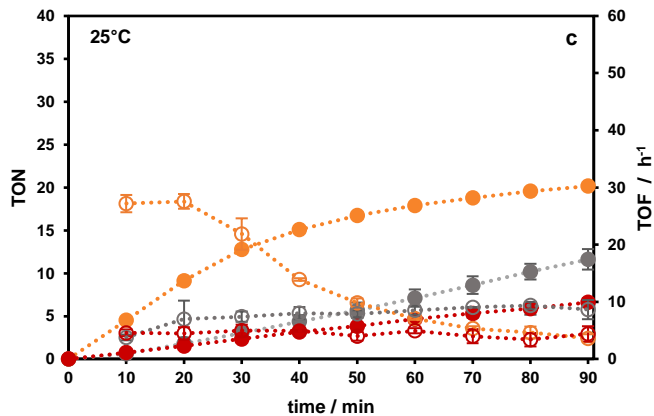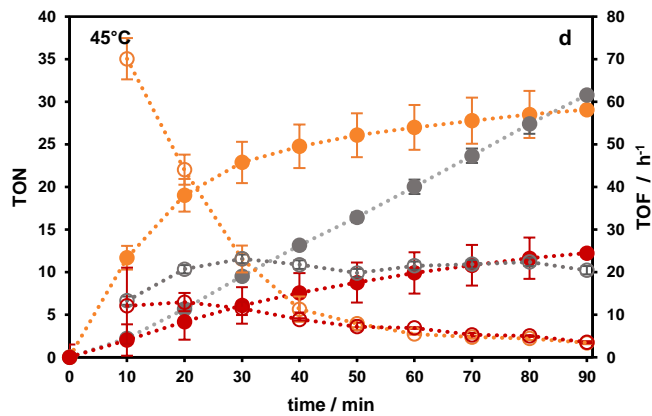

Supplement: Supplementary file 4 — Source Data [file 41467_2022_30147_MOESM4_ESM.zip › SourceData/Source Data Fig. 7/figure_7.pdf]

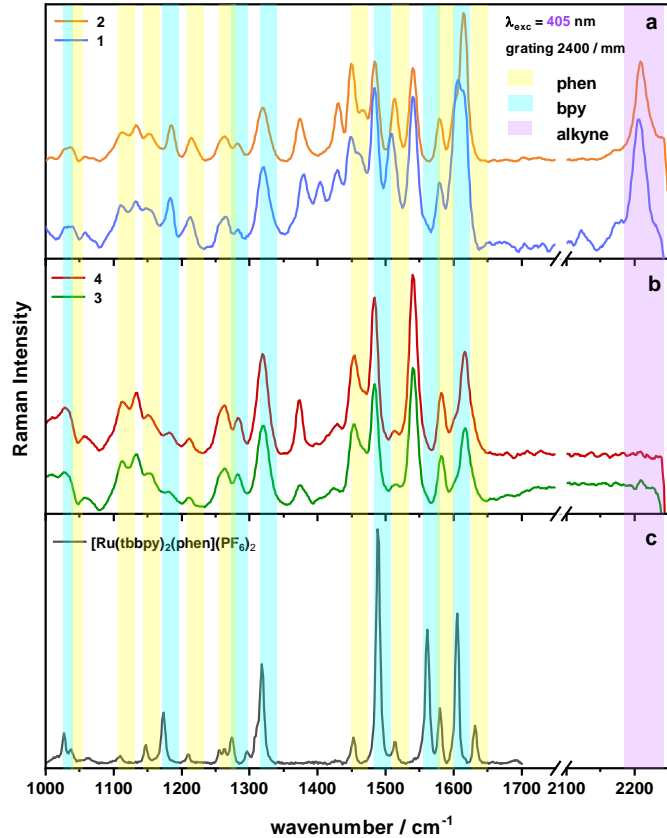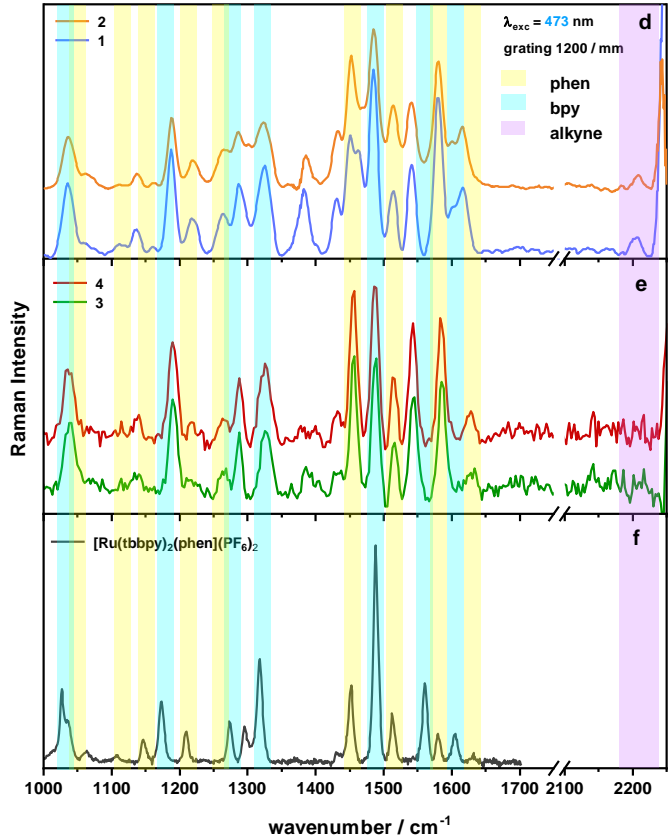

Supplement: Supplementary file 4 — Source Data [file 41467_2022_30147_MOESM4_ESM.zip › SourceData/Source Data Fig. 8/figure_8.pdf]

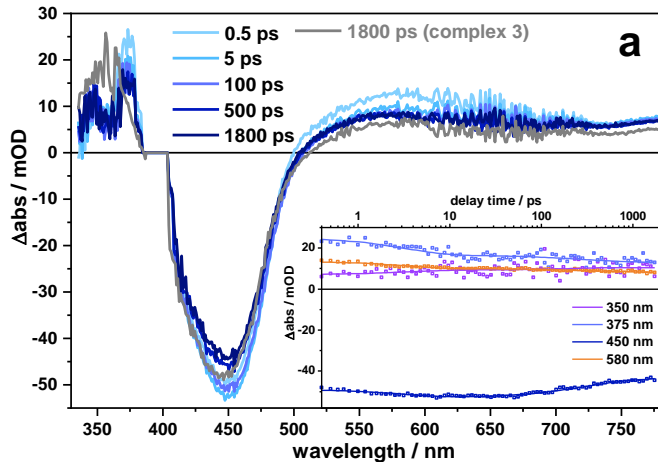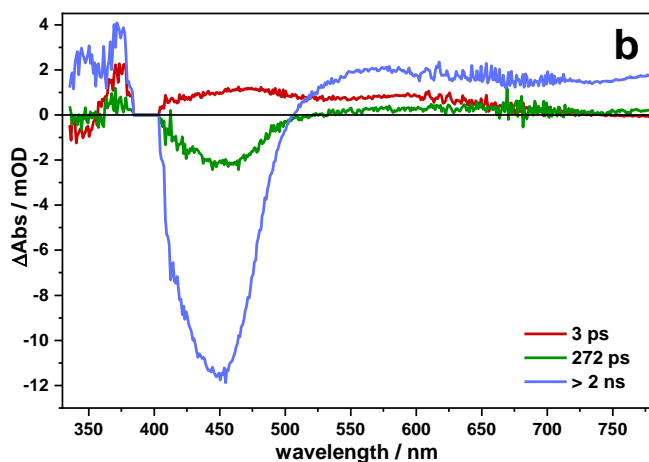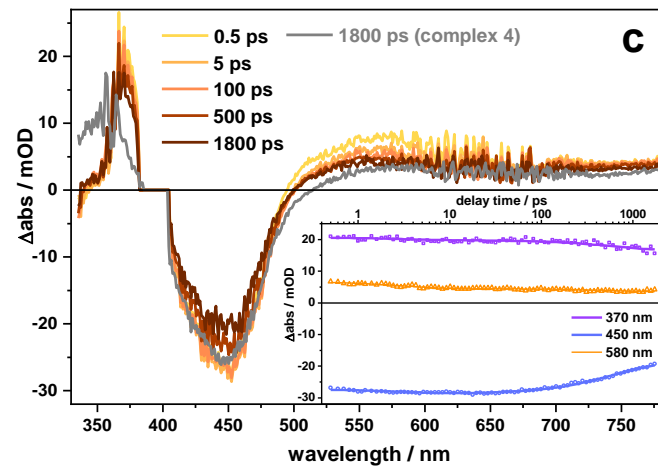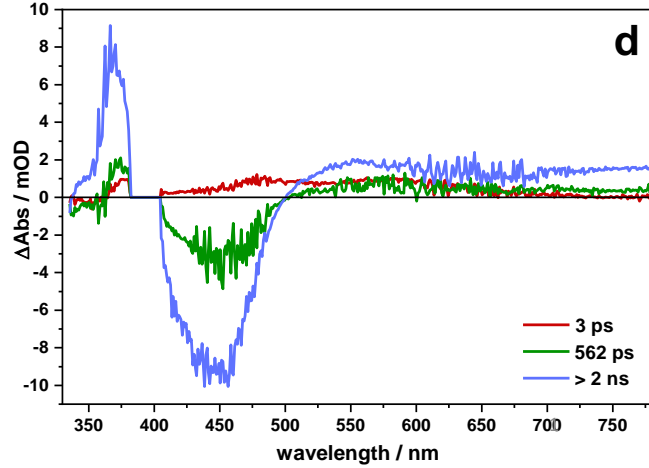

Supplement: Supplementary file 4 — Source Data [file 41467_2022_30147_MOESM4_ESM.zip › SourceData/Source Data Fig. 9/figure_9.pdf]
